# Rapalogs downmodulate intrinsic immunity and promote cell entry of SARS-CoV-2

**DOI:** 10.1101/2021.04.15.440067

**Published:** 2021-04-16

**Authors:** Guoli Shi, Abhilash I. Chiramel, Saliha Majdoul, Kin Kui Lai, Sudipto Das, Paul A. Beare, Thorkell Andresson, Sonja M. Best, Alex A. Compton

**Affiliations:** 1 HIV Dynamics and Replication Program, Center for Cancer Research, National Cancer Institute, Frederick, MD 21702; 2 Laboratory of Virology, Rocky Mountain Laboratories, National Institute of Allergy and Infectious Diseases, Hamilton, MT 59840; 3 Protein Characterization Laboratory, Center for Cancer Research, National Cancer Institute, Frederick, MD 21702

**Keywords:** rapamycin, rapalog, mTOR inhibitor, IFITM, interferon, SARS, COVID-19, coronavirus, Influenza, membrane fusion

## Abstract

Infection by SARS-CoV-2 generally causes mild symptoms but can lead to severe disease and death in certain populations, including the immunocompromised. Drug repurposing efforts are underway to identify compounds that interfere with SARS-CoV-2 replication or the immunopathology it can elicit. Rapamycin is among those being currently tested in clinical trials for impacts on COVID-19 severity. While rapamycin and rapamycin analogs (rapalogs) are FDA-approved for use as mTOR inhibitors in multiple clinical settings, including cancer, we previously found that rapamycin can increase the susceptibility of cells to infection by Influenza A virus. In this study, we tested the impact of rapalogs on cellular susceptibility to SARS-CoV-2 infection. We report that rapamycin and rapalogs increased SARS-CoV-2 titers in human cervical epithelial and lung epithelial cell lines to different extents, and a similar pattern of enhancement was observed using pseudovirus incorporating viral fusion proteins from SARS-CoV-2, SARS-CoV, MERS, and Influenza A Virus. Rapalogs also promoted cell entry driven by SARS-CoV-2 Spike in nasal cells and primary small airway cells, representing proximal and distal ends of the human respiratory tract, respectively. Interestingly, cell entry enhancement by the rapalog ridaforolimus was cell type-dependent, revealing a previously unrecognized functional divergence between rapalogs. The differential activity of rapalogs was associated with their capacity to induce the degradation of interferon-inducible transmembrane (IFITM) proteins, restriction factors that broadly inhibit virus infection. Our findings will spur the development of mTOR inhibitors that do not suppress the cell’s first line of antiviral defense.

## Introduction

Severe acute respiratory syndrome (SARS) coronavirus (CoV)-2 emerged in humans in 2019 following a species jump from bats and a possible intermediate animal host and is the cause of COVID-19, a respiratory and multi-organ disease of variable severity [1, 2]. The characterization of virus-host interactions that dictate SARS-CoV-2 infection and COVID-19 severity is a major priority for public health [3]. Basic research aimed at identifying the cellular factors required for SARS-CoV-2 infection (host dependency factors) has provided targets for antiviral drug development or repurposing [4–10]. In parallel, the identification of cellular factors capable of inhibiting SARS-CoV-2 replication (host restriction factors) has revealed intrinsic antiviral defenses that should be maintained and bolstered in susceptible individuals, especially those immunocompromised by age, immunodeficiency, or medical regimen [11–13]. Immune impairment, such as that resulting from cancer, has been associated with prolonged SARS-CoV-2 shedding and the seeding of “super-spreader” events [14–18]. As we consider host-directed small molecules as candidates for antiviral therapies, it remains critically important to assess their impact on both dependency factors and restriction factors before they are deployed to slow virus transmission and/or prevent viral pathogenesis in humans.

**Figure 1:**
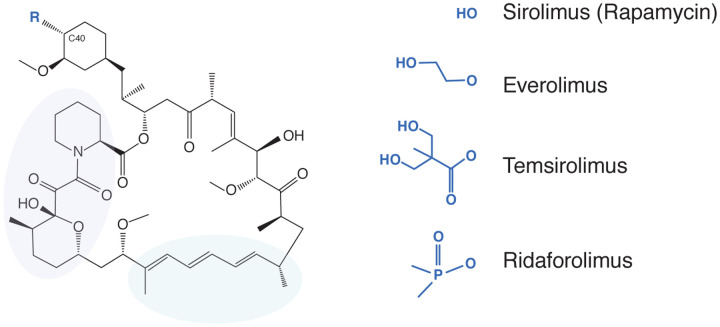
Rapamycin analogs share the same macrolide structure but differ by the functional group present at carbon-40. Violet and green bubbles indicate the FKBP12- and mTOR-binding sites, respectively.

One group of compounds being considered for the prevention and treatment of COVID-19-related immunopathology are rapamycin (Rapamune, sirolimus) and rapamycin analogs (rapalogs) [19–30]. As Food and Drug Administration-approved inhibitors of mammalian target of rapamycin (mTOR) kinase, these drugs are used therapeutically as immunosuppressants to inhibit the processes of cancer, autoimmunity, graft versus host disease, atherosclerosis, and aging [31]. Rapalogs, including everolimus (RAD-001), temsirolimus (Torisel, CCI-779), and ridaforolimus (AP-23573), were developed to alter the bioavailability and half-life of rapamycin for improved pharmacodynamics *in vivo* [32–36]. Differing by only a single functional group appended to carbon-40 of rapamycin (Figure 1), it is believed that rapamycin and rapalogs share the same molecular mechanism of action to inhibit mTOR [35]. Rapamycin binds to FK506-binding protein 12 (FKBP12) and the resulting complex physically interacts with mTOR and disrupts its signaling potential.

Activation of mTOR promotes cell growth, cell proliferation, and cell survival [37]. In addition, mTOR activation promotes pro-inflammatory T-cell differentiation and mTOR inhibitors have been used classically to block lymphocyte proliferation and cytokine storm [38]. Since respiratory virus infections can cause disease by provoking hyper-inflammatory immune responses that result in immunopathology, rapalogs are being tested as treatments to decrease viral disease burden. At least three active clinical trials have been designed to test the impact of rapamycin on COVID-19 severity (NCT04461340, NCT04341675, NCT04371640).

In addition to their potential utility for mitigating disease in individuals already infected by SARS-CoV-2, there are also calls to explore the use of rapalogs as antiviral agents to prevent infection itself [39]. It was previously reported that mTOR activation promotes infection by the human coronavirus that causes Middle East Respiratory Syndrome (MERS-CoV) [40]. However, the relationship between mTOR and virus replication is complex, varies from virus to virus, and is dynamic according to a given step in the virus life cycle [41–43].

Rapamycin and temsirolimus have been shown to promote Influenza A replication in mice and to exacerbate viral disease [44, 45]. Furthermore, we recently demonstrated that acute rapamycin treatment increases cellular susceptibility Influenza A virus infection by inducing the selective degradation of interferon-inducible transmembrane (IFITM) proteins [46]. IFITM proteins are expressed basally in a variety of tissues and are important components of cell-intrinsic immunity, the antiviral network that defends individual cells against virus invasion [47, 48]. We showed that rapamycin triggers the sorting of endosomal IFITM2 and IFITM3 into lysosomes for proteolytic degradation through a process that was independent of macroautophagy yet dependent on ubiquitin and endosomal complexes required for transport (ESCRT) machinery [46]. These findings revealed a previously unrecognized immunosuppressive feature of rapamycin acting on intrinsic immunity. Since IFITM proteins broadly inhibit the cellular entry of diverse enveloped viruses by disfavoring virus-cell membrane fusion in endosomes and at the plasma membrane [49–54], rapamycin exposure may increase cellular susceptibility to infection by many viral pathogens. We and others have previously shown that SARS-CoV-2 infection can be inhibited by ectopic and endogenous IFITM1, IFITM2, and/or IFITM3 in cell types that are permissive to infection [11, 13, 55, 56]. Additionally, single nucleotide polymorphisms in *IFITM3* known as ns12252 and rs34481144, previously associated with severe outcomes in Influenza A infection, have been associated with severe COVID-19 [57, 58]. Therefore, we tested the impact of rapamycin and rapalogs (hereby collectively referred to as rapalogs) on SARS-CoV-2 infection and characterized the stages of infection affected.

## Results

**Figure 2:**
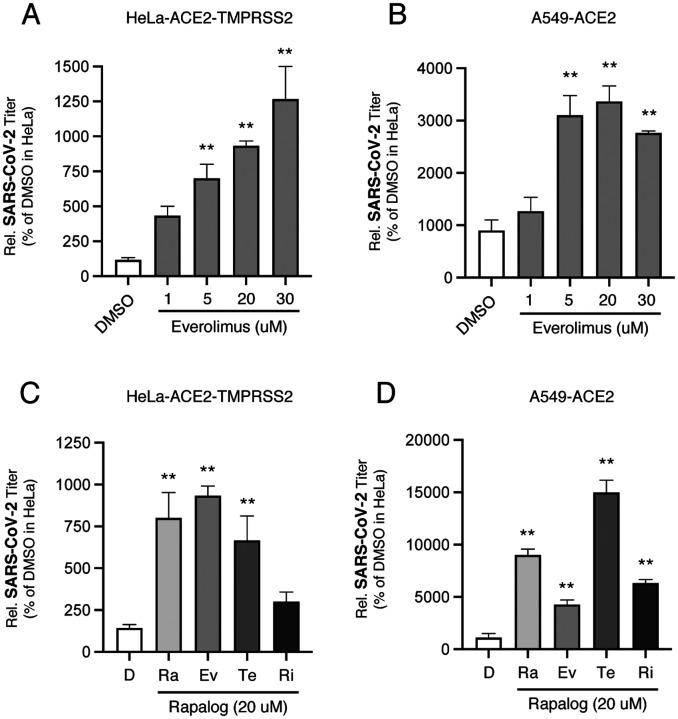
Rapalogs promote SARS-CoV-2 infection in cervical and lung epithelial cell lines in a dose-dependent and drug-dependent manner. (A) HeLa-ACE2-TMPRSS2 cells were treated with increasing concentrations of everolimus for 4 hours and subsequently exposed to SARS-CoV-2 at an MOI of 0.1. A volume of DMSO corresponding to the highest dose of everolimus served as vehicle control. Virus-containing supernatants were harvested at 24 hours and titered on Vero E6 cells by plaque assay and plaque-forming units (pfu) per mL was normalized to 100 in the DMSO condition. (B) A549-ACE2 cells were treated as in (A) and pfu per mL was normalized to the DMSO condition in HeLa-ACE2-TMPRSS2 cells. (C) HeLa-ACE2-TMPRSS2 cells were treated with 20 μM of rapamycin (Ra), everolimus (Ev), temsirolimus (Te), ridaforolimus (Ri), or an equivalent volume of DMSO (D) for 4 hours and subsequently exposed to SARS-CoV-2 at an MOI of 0.1. Virus-containing supernatants were harvested at 24 hours and titered on Vero E6 cells by plaque assay and pfu per mL was normalized to 100 in the DMSO condition. (D) A549-ACE2 cells were treated as in (C) and pfu per mL was normalized to the DMSO condition in HeLa-ACE2-TMPRSS2 cells. All error bars represent standard error calculated from 3–5 experiments. Statistical analysis was performed with one-way ANOVA and asterisks indicate significant difference from DMSO. *, p < 0.05; **, p < 0.01.

Two cell lines, cervical epithelial cells transduced with human ACE2 and TMPRSS2 (HeLa-ACE2-TMPRSS2) and lung epithelial cells transduced with human ACE2 (A549-ACE2), were pretreated with everolimus at varying concentrations for four hours prior to addition of SARS-CoV-2. Cell culture media was harvested 24 hours after virus addition and infectious virus titers were measured on Vero cells by plaque assay. We found that everolimus enhanced virus titers in a dose dependent manner, with a 20 micromolar dose increasing infectious yields by 8-fold in HeLa-ACE2-TMPRSS2 (Figure 2A) and by 3.5-fold in A549-ACE2 (Figure 2B). The effect of everolimus relative to other rapalogs was assessed by pretreating cells with 20 micromolar amounts for four hours followed by addition of SARS-CoV-2. In HeLa-ACE2-TMPRSS2 cells, rapamycin and temsirolimus promoted infection to a similar extent as everolimus, while the effect of ridaforolimus was negligible (Figure 2C). In A549-ACE2 cells, rapamycin and temsirolimus enhanced infectious yield by 9- and 15-fold, respectively, while everolimus and ridaforolimus did so to a smaller degree (Figure 2D). There results indicate that rapalogs generally increase the infectious yield of SARS-CoV-2 from cells.

**Figure 3:**
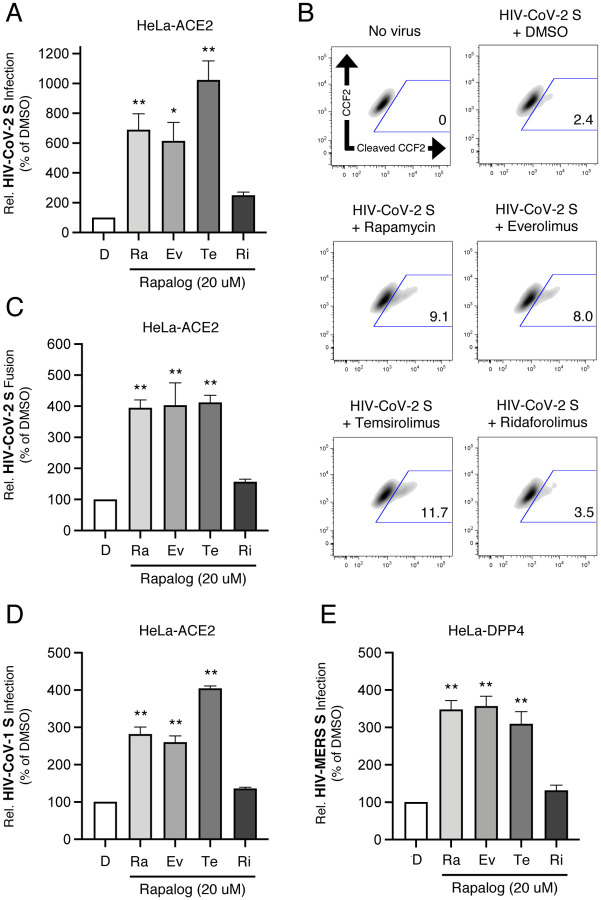
Rapalogs boost the cell entry step of infection mediated by Spike proteins from SARS-CoV-2, SARS-CoV, and MERS. (A) HeLa-ACE2 cells were treated with 20 μM of rapamycin (Ra), everolimus (Ev), temsirolimus (Te), ridaforolimus (Ri), or an equivalent volume of DMSO (D) for 4 hours and subsequently exposed to HIV-CoV-2 S pseudovirus. Cells were lysed at 48 hours and luciferase activity was measured. Luciferase units were normalized to 100 in the DMSO condition. (B) HeLa-ACE2 cells were treated with 20 μM of rapamycin (Ra), everolimus (Ev), temsirolimus (Te), ridaforolimus (Ri), or an equivalent volume of DMSO (D) for 4 hours and subsequently exposed to HIV-CoV-2 S pseudovirus incorporating BlaM-Vpr for 2 hours. Cells were incubated with CCF2-AM for an additional 2 hours and fixed. Cleaved CCF2 was measured by flow cytometry. Dot plots visualized as density plots from one representative experiment are shown and the percentage of CCF2+ cells which exhibit CCF2 cleavage is indicated. (C) Summary data for (B) representing the average of three experiments. (D) As in (A), except cells were exposed to HIV-CoV S pseudovirus. (E) As in (A), except HeLa-DPP4 cells were exposed to HIV-MERS S pseudovirus. All error bars represent standard error calculated from 3–4 experiments. Statistical analysis was performed with one-way ANOVA and asterisks indicate significant difference from DMSO. *, p < 0.05; **, p < 0.01.

To identify the stage of virus replication that is promoted by rapalogs, we took advantage of a pseudovirus system based on human immunodeficiency virus. This pseudovirus (HIV-CoV-2 S) is limited to a single round of infection, cell entry is mediated by SARS-CoV-2 Spike, and infection of target cells is measured by luciferase activity. Rapalogs were tested for activity on HIV-CoV-2 S in HeLa-ACE2 cells, since, in contrast to SARS-CoV-2, this pseudovirus does not require TMPRSS2 for cell entry. Rapamycin, everolimus, and temsirolimus enhanced HIV-CoV-2 S infection by up to an order to magnitude, while ridaforolimus had little effect (Figure 3A). These results approximate those obtained using replication-competent SARS-CoV-2, revealing that rapalogs promote the Spike-mediated cell entry step in a drug-dependent manner. We also tested the effect of tacrolimus (FK506), a compound with a chemical structure nearly identical to that of the rapalogs but which does not bind mTOR. We found that tacrolimus did not promote infection of HeLa-ACE2 by HIV-CoV-2 S (Supplemental Figure 1A).

Virus entry into cells is a concerted process involving virus attachment to the cell surface followed by fusion of cellular and viral membranes. Therefore, we carried out a virus-cell fusion assay in order to quantify the terminal stage of virus entry into single cells using flow cytometry. We found that treatment of cells with rapamycin, everolimus, and temsirolimus resulted in enhanced virus-cell fusion while ridaforolimus had no such effect (Figure 3B and 3C). These findings suggest that rapalogs promote the cell entry process mediated by SARS-CoV-2 Spike by facilitating virus-cell membrane merger. To measure whether rapalogs promote the cell entry process driven by other coronavirus Spike proteins, we produced HIV incorporating Spike from SARS-CoV (HIV-CoV S) or MERS (HIV-MERS S). Infections by both HIV-CoV S and HIV-MERS S were elevated by rapalog treatment of HeLa-ACE2 and HeLa-DPP4, respectively, although the extent of enhancement was lower than that observed with HIV-CoV-2 S (Figure 3D and 3E). Consistently, ridaforolimus was the least active among the rapalogs tested and it did not significantly promote pseudovirus infection. Since we previously showed that rapamycin promotes the cell entry process of Influenza A virus, we also assessed infection of pseudovirus incorporating hemagglutinin (HIV-HA). HA-mediated infection was enhanced 10-fold by rapamycin and everolimus, while temsirolimus enhanced by more than 30-fold. In contrast, ridaforolimus was inactive (Supplemental Figure 1B).

**Figure 4:**
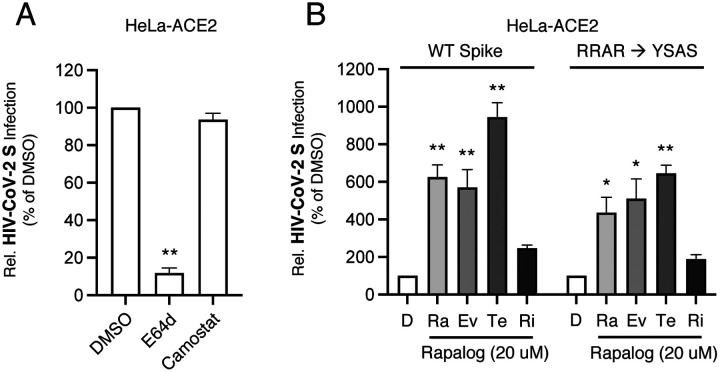
Rapalogs facilitate endocytic entry of HIV-CoV-2 S pseudovirus into HeLa-ACE2 cells. (A) HeLa-ACE2 cells were treated with 25 μM of E64d, camostat mesylate, or an equivalent volume of DMSO for 2 hours and subsequently exposed to HIV-CoV-2 S pseudovirus. At 48 hours, cells were lysed and luciferase activity was measured. Luciferase units were normalized to 100 in the DMSO condition. (B) HeLa-ACE2 cells were treated with 20 μM of rapamycin (Ra), everolimus (Ev), temsirolimus (Te), ridaforolimus (Ri), or an equivalent volume of DMSO (D) for 4 hours and subsequently exposed to HIV-CoV-2 S pseudovirus bearing WT or _682_YSAS_685_ Spike. Cells were lysed at 48 hours and luciferase activity was measured. Luciferase units were normalized to 100 in the DMSO condition. All error bars represent standard error calculated from 3 experiments. Statistical analysis was performed with one-way ANOVA and asterisks indicate significant difference from DMSO. *, p < 0.05; **, p < 0.01.

SARS-CoV-2 can enter cells via multiple routes, and sequential proteolytic processing of Spike is essential to this process. SARS-CoV-2 Spike is cleaved at a polybasic motif (RRAR) located at the S1/S2 boundary by furin-like proteases in virus-producing cells prior to release. Subsequently, the S2’ site is cleaved by trypsin-like proteases such as TMPRSS2 on the target cell surface or cathepsins B and L in target cell endosomes, activating the viral fusion peptide and facilitating membrane fusion [59–61]. It was previously shown that deletion or mutation of the polybasic RRAR motif in Spike promotes the endocytic, cathepsin-dependent entry pathway [4, 56], as has been shown for MERS Spike [62]. We found that inhibition of cathepsin activity by E64d blocked pseudovirus infection in HeLa-ACE2 cells, while inhibition of TMPRSS2 activity by camostat mesylate had little to no effect (Figure 4A), supporting the notion that an endosomal entry pathway is predominantly used by SARS-CoV-2 Spike in these cells [63]. To directly test whether rapalogs enhance Spike-mediated entry at the plasma membrane or in endosomes, we produced pseudovirus with Spike mutated at the polybasic _682_RRAR_685_ motif (RRAR → YSAS). We confirmed that introduction of YSAS prevented cleavage of Spike into S1 and S2 subunits in virus-producing cells (Supplemental Figure 2). Using pseudovirus incorporating mutant spike, we found that the capacity for select rapalogs to promote infection was mostly intact (Figure 4B). These results suggest that rapalog treatment facilitates endosomal Spike-mediated entry in HeLa-ACE2 cells.

**Figure 5:**
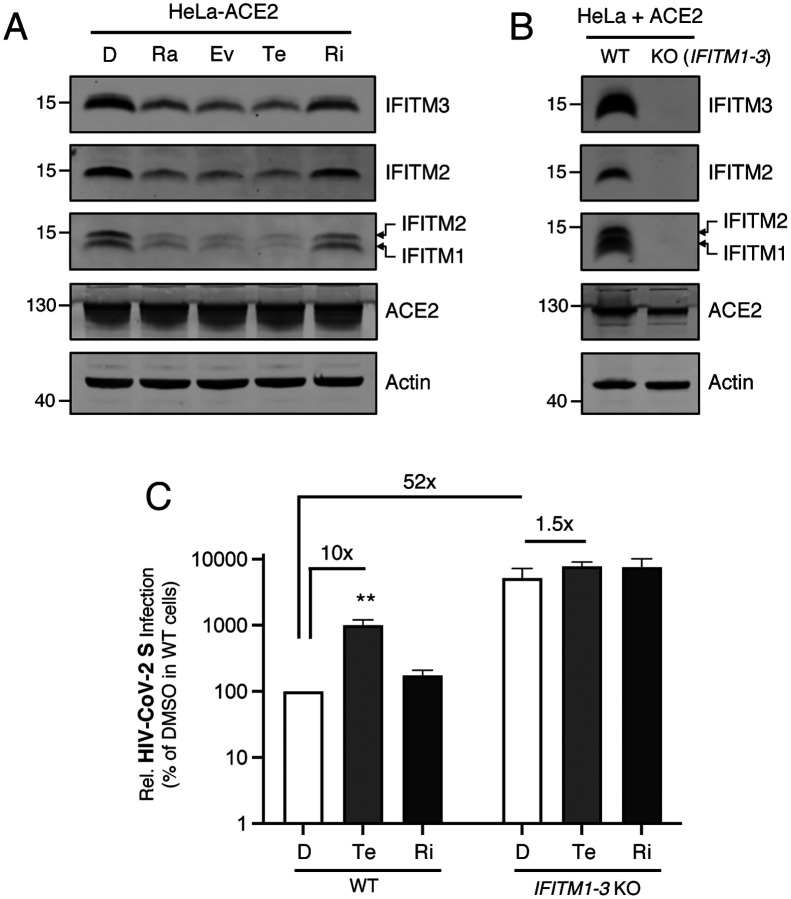
Human IFITM proteins restrict Spike-mediated infection and their levels are decreased differentially by rapalogs. (A) HeLa-ACE2 cells were treated with 20 μM of rapamycin (Ra), everolimus (Ev), temsirolimus (Te), ridaforolimus (Ri), or an equivalent volume of DMSO (D) for 4 hours and whole cell lysates were subjected to SDS-PAGE and Western blot analysis. Immunoblotting was performed with anti-IFITM2, anti-IFITM3, anti-IFITM1, anti-ACE2, and anti-actin (in that order) on the same nitrocellulose membrane. (B) Whole cell lysates from HeLa and HeLa *IFITM1–3* KO cells transiently transfected with ACE2 were subjected to SDS-PAGE and Western blot analysis. Immunoblotting was performed with anti-IFITM2, anti-IFITM3, anti-IFITM1, anti-ACE2, and anti-actin (in that order) on the same nitrocellulose membrane. Numbers and tick marks indicate size (kilodaltons) and position of protein standards in ladder. (C) HeLa and HeLa *IFITM1–3* KO cells were transiently transfected with ACE2 for 24 hours and subsequently treated with 20 μM of temsirolimus (Te), ridaforolimus (Ri), or an equivalent volume of DMSO (D) for 4 hours. Cells were exposed to HIV-CoV-2 S pseudovirus and luciferase activity was measured 48 hours later. Luciferase units were normalized to 100 in the DMSO condition of WT cells. All error bars represent standard error calculated from 4 experiments. Statistical analysis was performed with one-way ANOVA and asterisks indicate significant difference from DMSO. *, p < 0.05; **, p < 0.01.

We previously demonstrated that mTOR inhibition by rapamycin triggers a rapid degradation of IFITM proteins in various cell types, including HeLa, primary human foreskin fibroblasts, and CD34+ hematopoietic stem cells [46]. To compare and contrast the effects of rapalogs on steady-state IFITM protein levels, we treated HeLa-ACE2 cells with rapamycin, everolimus, temsirolimus, or ridaforolimus for four hours. We found that everolimus and temsirolimus treatment reduced levels of IFITM1, IFITM2, and IFITM3 proteins to a similar extent as rapamycin, while ACE2 levels were unaffected. However, ridaforolimus resulted in only a modest reduction of IFITM proteins (Figure 5A). Rapalogs promote the proteolytic degradation of IFITM proteins in endolysosomes, since this process was inhibited by bafilomycin A1 (Supplemental Figure 3). To formally address how rapalog-mediated depletion of IFITM proteins impacts SARS-CoV-2 Spike-mediated entry, we used HeLa cells in which IFITM1, IFITM2, and IFITM3 are knocked out (*IFITM1–3* KO) and introduced human ACE2 by transient transfection (Figure 5B). Relative to WT cells, where temsirolimus promotes infection by approximately 10-fold, the enhancement effect was reduced to 1.5-fold in *IFITM1–3* KO cells. Furthermore, pseudovirus infection was approximately 50-fold higher in *IFITM1–3* KO cells. These findings indicate that endogenous IFITM1, IFITM2, and IFITM3 substantially restrict SARS-CoV-2 Spike-mediated infection in HeLa-ACE2 cells and that rapalogs promote infection by lowering their levels in cells. In order to better characterize the degradation program triggered by rapalogs and to identify cellular proteins other than IFITM that are modulated following rapalog treatment, we performed proteomics by mass spectrometry on HeLa-ACE2 cells treated with DMSO, temsirolimus, or ridaforolimus. The complete list of over 7000 proteins detected is available in Supplemental File 1. While the IFITM proteins themselves were not detected in our analysis, we identified a select number of additional proteins whose levels were decreased significantly by rapamycin and/or temsirolimus and, to a lesser extent, by ridaforolimus (Supplemental Figure 4).

**Figure 6:**
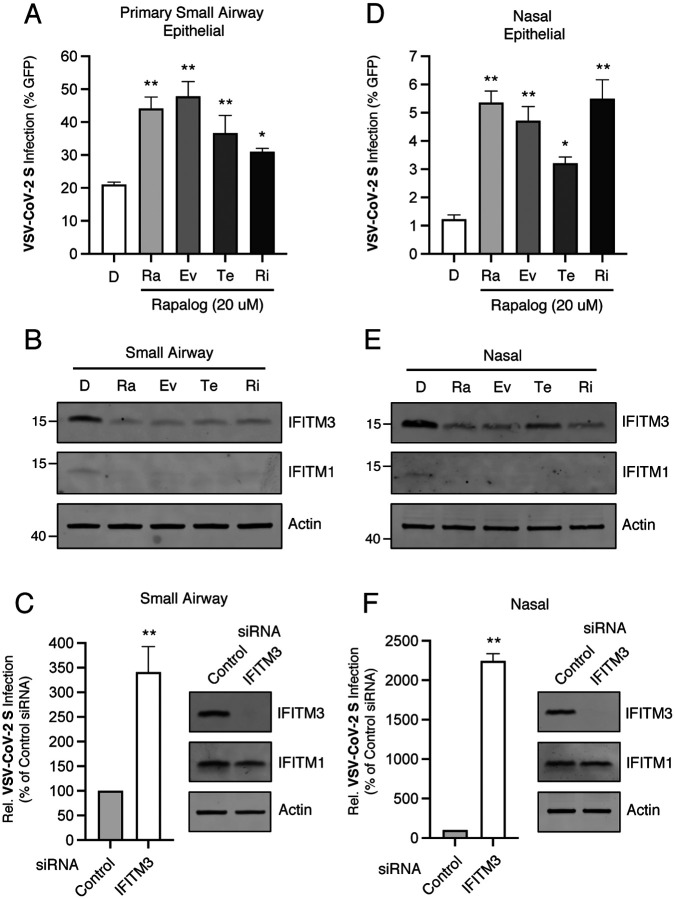
Rapalogs promote infection mediated by SARS-CoV-2 Spike in primary lung and nasal cells. (A) Primary human small airway epithelial cells were treated with 20 μM of rapamycin (Ra), everolimus (Ev), temsirolimus (Te), ridaforolimus (Ri), or an equivalent volume of DMSO (D) for 4 hours and subsequently exposed to VSV-CoV-2 S pseudovirus. Cells were fixed at 24 hours and infection was scored by measuring GFP+ cells with flow cytometry. (B) Whole cell lysates from primary small airway epithelial cells were subjected to SDS-PAGE and Western blot analysis. Immunoblotting was performed with anti-IFITM2, anti-IFITM3, anti-IFITM1, and anti-actin (in that order) on the same nitrocellulose membrane. (C) Primary human small airway epithelial cells were transfected with siRNA targeting IFITM3 or a non-targeting control siRNA and exposed to VSV-CoV-2 S pseudovirus 48 hours later. At 24 hours post-virus addition, cells were lysed and luciferase activity was measured. Luciferase units were normalized to 100 in the control siRNA condition. siRNA-transfected cells were subjected to SDS-PAGE and Western blot analysis with anti-IFITM2, anti-IFITM3, anti-IFITM1, and anti-actin (in that order) on the same nitrocellulose membrane. (D) Semi-transformed human nasal epithelial cells were treated with 20 μM of rapamycin (Ra), everolimus (Ev), temsirolimus (Te), ridaforolimus (Ri), or an equivalent volume of DMSO (D) for 4 hours and subsequently exposed to VSV-CoV-2 S pseudovirus. Cells were fixed at 24 hours and infection was scored by measuring GFP+ cells with flow cytometry. (E) Whole cell lysates from human nasal epithelial cells were subjected to SDS-PAGE and Western blot analysis. Immunoblotting was performed with anti-IFITM2, anti-IFITM3, anti-IFITM1, and anti-actin (in that order) on the same nitrocellulose membrane. (F) Human nasal epithelial cells were transfected with 20 nM siRNA targeting IFITM3 or a non-targeting control siRNA and exposed to VSV-CoV-2 S pseudovirus 48 hours later. At 24 hours post-virus addition, cells were lysed and luciferase activity was measured. Luciferase units were normalized to 100 in the control siRNA condition. siRNA-transfected cells were subjected to SDS-PAGE and Western blot analysis with anti-IFITM2, anti-IFITM3, anti-IFITM1, and anti-actin (in that order) on the same nitrocellulose membrane. Numbers and tick marks indicate size (kilodaltons) and position of protein standards in ladder. All error bars represent standard error calculated from 3–4 experiments. Statistical analysis in (A) and (D) was performed with one-way ANOVA and asterisks indicate significant difference from DMSO. Statistical analysis in (C) and (F) was performed with Student’s T test and asterisks indicate significant difference from control siRNA. *, p < 0.05; **, p < 0.01.

To explore the impact of rapalogs on SARS-CoV-2 infection in additional cell types that model the human respiratory tract, we used primary human small airway (lung) epithelial cells (herein referred to as small airway cells) and partially transformed human nasal epithelial cells (UNCNN2TS, herein referred to as nasal cells) [64, 65]. These cells were not permissive to HIV-CoV-2 S pseudovirus, but we were able to infect them with a pseudovirus based on vesicular stomatitis virus (VSV-CoV-2 S) [66]. We found that small airway cells were highly permissive to VSV-CoV-2 S and that rapalogs promoted infection to different extents. Specifically, rapamycin, everolimus, and temsirolimus enhanced infection by up to 2.5-fold while ridaforolimus was less active (Figure 6A). In order to draw parallels between the mechanism of action of rapalogs on different cell types, we assessed the basal levels of IFITM proteins in small airway cells and tested whether their levels were altered by the drugs. Endogenous IFITM3 protein was detected and was reduced to different extents by rapalogs, while IFITM1 was found at lower levels and IFITM2 was not detected at all (Figure 6B). We then asked whether IFITM3 in small airway cells was consequential for VSV-CoV-2 S infection by using RNA interference. Interestingly, transfection of control siRNA resulted in increased IFITM1 protein relative to basal levels, but not that of IFITM2, and specific knockdown of IFITM3 resulted in a more than 3-fold enhancement of infection (Figure 6C). These results suggest that rapalogs promote SARS-CoV-2 Spike-mediated infection in small airway cells, at least in part, by downmodulating IFITM3. We performed a similar series of experiments in nasal cells and found that, while these cells were relatively resistant to VSV-CoV-2 S pseudovirus, infection levels were elevated in the presence of rapalogs (Figure 6D). Here, rapamycin, everolimus, and ridaforolimus treatment resulted in higher levels of infection compared to temsirolimus treatment, indicating that the bioactivity of some rapalogs is cell-type dependent. When we examined the extent to which endogenous IFITM proteins were modulated by the drugs in nasal cells, we found that IFITM3 was abundantly expressed and was differentially downmodulated by rapalogs, with temsirolimus exhibiting the lowest capacity to decrease IFITM3 levels. In these cells, IFITM1 was barely detectable and IFITM2 was not detected (Figure 6E). As seen in small airway cells, siRNA transfection induced IFITM1 protein in nasal cells. Upon specific knockdown of IFITM3, infection was enhanced by more than 20-fold (Figure 6F). Overall, these data support a model whereby rapalog treatment of cells modeling the human respiratory tract results in drug-dependent degradation of IFITM proteins and increased susceptibility to SARS-CoV-2 Spike-mediated infection.

## Discussion

We previously demonstrated that treatment of cells with micromolar quantities of rapamycin induces the lysosomal degradation of IFITM proteins via a pathway that is independent of macroautophagy but dependent upon ESCRT-mediated sorting of IFITM proteins into intraluminal vesicles of late endosomes [46]. Our understanding of how this degradation pathway is mediated is incomplete, but it likely results from a form of non-canonical autophagy known as microautophagy [67]. One type of microautophagy that has been described in yeast and mammalian cells is characterized by ESCRT-dependent sorting of endolysosomal membrane proteins into intraluminal vesicles followed by their degradation by lysosomal hydrolases. Furthermore, there is evidence indicating that microautophagy is regulated by mTOR [68]. It was recently shown in yeast that mTOR inhibition triggers a ubiquitin- and ESCRT-dependent turnover of vacuolar (lysosomal) membrane proteins [69, 70]. Apart from our immunoblotting of IFITM proteins, our proteomics analysis suggests that rapalogs differentially induce a program that turns over a select group of cellular proteins (1% or less of proteins detected were reduced by 40% or more following treatment with any rapalog). Ongoing work will identify whether these proteins are co-regulated directly or indirectly by the same E3 ubiquitin ligase, and whether this ligase is controlled by mTOR activity.

IFITM2 and IFITM3 are cell surface proteins which are targeted to endolysosomes via a tyrosine-based endocytic motif (YxxL) in the amino terminus, and phosphorylation regulates this process [71–75]. In contrast, it is often cited that IFITM1, which lacks the YxxL motif, is primarily localized to the plasma membrane of cells. However, the carboxy-terminus of IFITM1 contains its own sorting signal and is responsible for a proportion of IFITM1 being localized to endosomes [76, 77]. Our previous efforts found that FLAG-tagged IFITM2 and IFITM3 were sensitive to rapamycin-induced degradation while FLAG-IFITM1 was not [46]. In the current study, we find that all three of the IFITM proteins, which are expressed at high basal levels in HeLa cells, are degraded following rapalog treatment. This likely reflects a partial overlap in endolysosomal localization between IFITM1, IFITM2, and IFITM3. In support of IFITM1 being partially localized to lysosomes is the fact that it inhibits the cell entry of Marburg and Ebola viruses, which require lysosomal cathepsins for the final step of viral fusion [78]. There remains much to be learned about the sequence determinants and cellular machinery required to carry out degradation of IFITM proteins during rapalog treatment. It was previously discovered that NEDD4 E3 ligase regulates the degradation of IFITM2 and IFITM3 [74, 79], but whether other E3 ligases mediate the regulation of IFITM proteins during mTOR inhibition is unknown.

In addition to the degradation of basal IFITM3 expressed at steady-state, we previously reported that rapamycin promotes the turnover of IFITM3 induced by type-I interferon [46]. While our results indicate that rapalogs differentially impact basal levels of IFITM proteins in multiple cell types, we did not assess how they affect those induced by infection. This would be required to fully understand how rapalogs promote cellular susceptibility to infection, and to what extent rapalog-mediated regulation of IFITM proteins is responsible. In contrast to HeLa cells, we did not detect IFITM1, IFITM2, or IFITM3 protein in A549 cells under basal conditions. Nonetheless, it has been shown that IFITM3 is among the most highly induced genes in primary human lung epithelial cells exposed to SARS-CoV-2 [82, 83]. Therefore, it is possible that cell type-specific patterns of virus-induced IFITM will need to be assessed in order to fully understand the effects of different rapalogs on cellular susceptibility to virus infection. Nonetheless, it is notable that we observed a relatively high level of basal IFITM3 present in nasal epithelial cells, and furthermore, that specific knockdown of IFITM3 resulted in a more than 20-fold gain in cellular susceptibility to SARS-CoV-2 Spike-mediated infection in this cell type. The upper airway, and the nasal epithelium in particular, likely represents the initial site of infection by SARS-CoV-2 [84]. It was reported that infected humans experiencing mild or moderative COVID-19 showed extensive induction of antiviral genes, including *IFITM1* and *IFITM3*, in their nasal epithelium compared to individuals suffering from more severe COVID-19 [85]. This finding suggests that cell-intrinsic immunity in the nasal epithelium plays a role in constraining virus-induced pathology in the lower respiratory tract. Taken together, these results suggest that rapalog use and its downmodulating effects on IFITM proteins may facilitate virus spread between humans. It would be interesting to examine whether any of the other proteins differentially downmodulated or upmodulated by rapalogs have an impact on SARS-CoV-2 infection in the various cell types studied here.

All of the rapamycin-derived compounds tested here are believed to act as allosteric mTOR inhibitors by promoting a tertiary complex consisting of drug, FKBP12, and mTOR. In contrast, tacrolimus (FK506) binds FKBP12 and calcineurin, but not mTOR [80]. The fact that tacrolimus does not enhance SARS-CoV-2 Spike-mediated infection suggests that mTOR inhibition is essential to this process. However, we also show that ridaforolimus enhances virus infection in HeLa and A549 cells to a lesser extent than the other rapalogs, which suggests that binding to mTOR and FKBP12 is insufficient to explain the phenotype. It is possible that enhancement of infection results from interactions between rapalogs and cellular proteins other than mTOR and FKBP12. Rapamycin has been shown to bind additional FKBPs, such as FKBP52, whose levels may vary between cell types [81]. Our results warrant an in-depth analysis of the cellular proteins directly bound by different rapalogs, and this should be carried out in different cell lines, since we show that the impact of ridaforolimus on infection is cell-type dependent.

Overall, and in combination with our previous work [46], these results draw attention to a novel immunosuppressive property of rapamycin and rapalogs that acts on intrinsic immunity and impacts cellular susceptibility to infection by multiple viruses, including SARS-CoV-2. Rapalogs are traditionally used for their immunosuppressive effects on adaptive immunity in order to mitigate systemic immunopathology, and this is one reason why they are being tested for therapeutic benefit in COVID-19 patients suffering from respiratory distress syndrome. Further work exploring how dosing of existing rapalogs in animal models impacts both the intended and unintended consequences of mTOR inhibition is a priority. Furthermore, the development of new mTOR inhibitors lacking this effect on intrinsic immunity are needed in order to minimize unintended immunocompromised states in humans.

## Materials and Methods

### Cell lines, cell culture, and inhibitors

HEK293T cells were obtained from ATCC (CRL-3216). HeLa-ACE2, HeLa-ACE2-TMPRSS2, HeLa-DPP4, and A549-ACE2 cell lines were produced by transducing cells with lentivirus packaging pWPI encoding ACE2, DPP4, or TMPRSS2 and selecting with blasticidin. HeLa IFITM1/2/3 Knockout (C5–9) cells were purchased from ATCC (CRL-3452). Primary human small airway (lung) epithelial cells (HSAEC) were purchased from ATCC (PCS-301–010). The partially-immortalized nasal epithelial cell line (UNCNN2TS) was kindly provided by Scott H. Randell (University of North Carolina School of Medicine). All cells were cultured at 37°C with 5% CO_2_ in Dulbecco’s Modified Eagle Medium (DMEM) supplemented with 10% fetal bovine serum (HyClone, Cytiva), except for UNCNN2TS, which were cultured in EpiX Medium (Propagenix), and HSAEC, which were cultured with airway epithelial cell basal medium (ATCC, PCS-300–030) and the bronchial epithelial cell growth kit (ATCC, PCS-300–040). Rapamycin (553211) was obtained from Sigma. Everolimus (S1120), temsirolimus (S1044), ridaforolimus (S5003), and tacrolimus (S5003) were obtained from Selleckchem. E64d (E8640) and camostat mesylate (SML0057) were obtained from Sigma.

### Plasmids and RNA interference

pcDNA3.1 encoding human ACE2 was kindly provided by Thomas Gallagher (Loyola University). pcDNA3.1 encoding CoV Spike or CoV-2 Spike tagged with a C9 epitope on the C-terminus, or MERS Spike, were kindly provided by Thomas Gallagher (Loyola University). pcDNA3.1 CoV-2 Spike encoding YSAS in the place of _682_RRAR_685_ was generated by site-directed mutagenesis. pcDNA3.1 encoding CoV Spike or CoV-2 Spike tagged with a FLAG epitope on the C-terminus, or VSV glycoprotein (VSV-G), were obtained from Michael Letko and Vincent Munster (NIAID). pWPI was obtained from Addgene (12254) and human ACE2 or human TMPRSS2 was introduced by Gateway cloning (Gateway LR Clonase II Enzyme mix (11791020)) as per manufacturer’s instructions. pPolII encoding hemagglutinin (HA) or neuraminidase (NA) from Influenza A/Turkey/1/2005 (H5N1) were kindly provided by Richard Yi Tsun Kao (The University of Hong Kong). pCMV encoding HIV-1 Vpr fused to beta lactamase (pCMV4-BlaMVpr) was obtained from ATCC (21950). pNL4–3LucR-E- was kindly provided by Vineet KewalRamani. pNL4–3E- was kindly provided by Olivier Schwartz (Institut Pasteur). Silence Select siRNA targeting IFITM3 (s195035) and a non-targeting control (No. 1) were obtained from Ambion. Cells were transfected with 20 nM siRNA using Opti-MEM (Gibco) and Lipofectamine RNAiMAX (Thermo Fisher).

### Virus and pseudovirus infections

SARS-CoV-2 strain nCoV-WA1–2020 (MN985325.1) was provided by the Centers for Disease Control. Virus propagation was performed in Vero E6 cells. Virus titers were calculated by plaque assay performed in Vero E6 cells as follows: Serial 10-fold dilutions were added to Vero E6 monolayers in 48-well plates for 1 hour at 37°C. Cells were overlayed with 1.5% carboxymethyl cellulose (Sigma) in modified Eagle’s medium containing 3% fetal bovine serum (Gibco), 1 mM L-glutamine, 50 units per mL penicillin and 50 μg per mL streptomycin. Three days post-infection, cells were fixed in 10% formalin and stained with crystal violet to visualize and count plaques as previously described [86]. Titers were calculated as plaque forming units per mL and normalized as described in the figure captions. HIV-based pseudovirus was produced by transfecting HEK293T cells with 12 μg of pNL4–3LucR-E- and 4 μg of pcDNA3.1 Spike (CoV, CoV-2, or MERS), or 2 μg of pPol1II HA and 2 μg of pPol1II NA using TransIT-293 (Mirus). Virus supernatant was harvested 72 hours post-transfection and filtered through 0.22 μm filters. Undiluted virus supernatant was added to target cells and incubated for 72 hours prior to lysis with Passive Lysis Buffer (Promega). Luciferase activity was measured using the Luciferase Assay System (Promega). VSV-based pseudovirus was produced as previously described [66]. In brief, HEK293T cells were transfected with 2 μg pcDNA3.1 and CoV, CoV-2, or MERS Spike using Lipofectamine2000 (Thermo Fisher). At 24 hours post-transfection, culture medium was removed from cells and 2 mL of VSV-luc/GFP + VSV-G was added. At 48 hours post-infection, virus supernatants were collected, clarified by centrifugation at 500×G for 5 mins, and stored. 50–200 μL of virus supernatants were added to target cells for a period of 24 hours prior to fixation with 4% paraformaldehyde (for measurements of GFP+ cells with flow cytometry) or lysis with Passive Lysis Buffer (for measurements of luciferase activity). E64d or camostat mesylate (25 μM) were used to pretreat cells for 2 hours prior to virus addition and were maintained for the duration of infection. For infections with replication-competent SARS-CoV-2, rapamycin, everolimus, temsirolimus, or ridaforolimus (20 μM) were used to pretreat cells for 4 hours and then drugs were washed away prior to addition of virus at a multiplicity of infection (MOI) of 0.1. DMSO (Sigma) was used as a vehicle control. At 1 hour post-virus addition, cells were washed once with 1X PBS and overlayed with complete medium. Supernatants were harvested 24 hours later and titers were determined on plaque assays performed in Vero E6 cells. For single-round infections using HIV- or VSV-based pseudovirus, rapamycin, everolimus, temsirolimus, ridaforolimus, or tacrolimus (20 μM) were used to pretreat cells for 4 hours and were maintained for the duration of infection and until harvest of cells for luciferase assay or flow cytometry. DMSO (Sigma) was used as a vehicle control.

### FRET-based virus-cell fusion assay

HIV-based pseudovirus incorporating BlaM-Vpr and CoV-2 Spike was produced by transfecting HEK293T cells with pNL4–3E- (15 μg), pCMV4-BlaM-Vpr (5 μg), and pcDNA3.1 CoV-2 Spike (5 μg) using the calcium phosphate technique. Briefly, six million 293T cells were seeded in a T75 flask. Plasmid DNA was mixed with sterile H_2_O, CaCl_2_, and Tris-EDTA (TE) buffer, and the totality was combined with Hepes-buffered saline (HBS). The transfection volume was added dropwise, and cells were incubated at 37°C for 48 h. Supernatants were clarified by centrifugation, passed through a 0.45 μm filter, and stored. Titers were measured using an HIV-1 p24 ELISA kit (XpressBio). 50 ng p25 equivalent of virus was added to HeLa-ACE2 cells for 2 hours. Cells were washed and labeled with the CCF2-AM β-lactamase Loading Kit (Invitrogen) for 2 hours and analyzed for virus-cell fusion by flow cytometry as described [87]. Rapamycin, everolimus, temsirolimus, or ridaforolimus (20 μM) were used to pretreat cells for 4 hours prior to virus addition and were maintained for the duration of infection. DMSO (Sigma) was used as a vehicle control.

### Western blot, flow cytometry, and antibodies

Whole cell lysis was performed with RIPA buffer (Thermo Fisher) supplemented with Halt Protease Inhibitor mixture EDTA-free (Thermo Fisher). Lysates were clarified by centrifugation and supernatants were collected and stored. Protein concentration was determined with the Protein Assay Kit II (Bio-Rad), and 10–15 μg of protein was loaded into 12% acrylamide Criterion XT Bis-Tris Precast Gels (Bio-Rad). Electrophoresis was performed with NuPage MES SDS Running Buffer (Invitrogren) and proteins were transferred to Amersham Protran Premium Nitrocellulose Membrane, pore size 0.20 μm (GE Healthcare). Membranes were blocked with Odyssey Blocking Buffer in PBS (Li-COR) and incubated with the following primary antibodies diluted in Odyssey Antibody Diluent (Li-COR): anti-IFITM1 (60074–1-Ig; Proteintech), anti-IFITM2 (66137–1-Ig; Proteintech), anti-IFITM3 (EPR5242, ab109429; Abcam), anti-actin (C4, sc-47778; Santa Cruz Biotechnology), anti-hACE2 (ab15348; Abcam), and anti-CoV Spike (PA1–41165; Thermo Fisher). Secondary antibodies conjugated to DyLight 800 or 680 (Li-Cor) and the Li-Cor Odyssey CLx imaging system were used to reveal specific protein detection. Images were analyzed and assembled using ImageStudioLite (Li-Cor).

### Proteomics by mass spectrometry

Protein Digestion and TMT labeling. Cell pellets were produced in triplicate from HeLa-ACE2 cells treated with 20 μM rapamycin, temsirolimus, ridaforolimus, or an equivalent volume of DMSO and lysed in 50 mM HEPES, pH 8.0 and 8M urea followed by sonication. Lysates were clarified by centrifugation and protein concentration was quantified using BCA protein estimation kit (Thermo Fisher). One hundred micrograms of lysate were alkylated and digested by addition of trypsin at a ratio of 1:50 (Promega) and incubating overnight at 37°C. Digestion was acidified by adding formic acid (FA) to a final concentration of 1% and desalted using peptide desalting columns (Thermo Fisher) according to manufacturer’s protocol. Peptides were eluted from the columns using 50% ACN/0.1% FA, dried in a speedvac, and kept frozen at −20°C until further analysis. For TMT labeling, 15 μg of each sample was reconstituted in 50 μL of 50 mM HEPES, pH 8.0, and 75 μg of TMTpro label (Thermo Fisher) in 100% ACN was added to each sample. After incubating the mixture for 1 hr at room temperature with occasional mixing, the reaction was terminated by adding 8 μL of 5% hydroxylamine. The peptide samples for each condition were pooled and cleaned using peptide desalting columns (Thermo Fisher). High pH reverse phase fractionation. The first dimensional separation of the peptides was performed using a Waters Acquity UPLC system coupled with a fluorescence detector (Waters) using a 150 mm × 3.0 mm Xbridge Peptide BEM^™^ 2.5 um C18 column (Waters) operating at 0.35 mL/min. The dried peptides were reconstituted in 100 μL of mobile phase A solvent (3 mM ammonium bicarbonate, pH 8.0). Mobile phase B was 100% acetonitrile (Thermo Fisher). The column was washed with mobile phase A for 10 min followed by gradient elution 0– 50% B (10–60 min) and 50–75 %B (60–70 min). The fractions were collected every minute. These 60 fractions were pooled into 24 fractions. The fractions were vacuum centrifuged to dryness and stored at −80°C until analysis by mass spectrometry. Mass Spectrometry acquisition and data analysis. The dried peptide fractions were reconstituted in 0.1% TFA and subjected to nanoflow liquid chromatography (Thermo Ultimate^™^ 3000RSLC nano LC system, Thermo Scientific) coupled to an Orbitrap Eclipse mass spectrometer (Thermo Scientific). Peptides were separated using a low pH gradient using 5–50% ACN over 120 minutes in mobile phase containing 0.1% formic acid at 300 nL/min flow rate. MS scans were performed in the Orbitrap analyser at a resolution of 120,000 with an ion accumulation target set at 4e^5^ and max IT set at 50ms over a mass range of 400–1600 m/z. Ions with determined charge states between 2 and 5 were selected for MS2 scans in the ion trap with CID fragmentation (Turbo; NCE 35%; maximum injection time 35 ms; AGC 1 × 10^4^). The spectra were searched using the Real Time Search Node in the tune file using human Uniprot database using Comet search algorithm with TMT16 plex (304.2071Da) set as a static modification of lysine and the N-termini of the peptide. Carbamidomethylation of cysteine residues (+57.0214 Da) was set as a static modification, while oxidation of methionine residues (+15.9949 Da) was set up as dynamic modification. For the selected peptide, an SPS–MS3 scan was performed using up to 10 *b*- and *y-*type fragment ions as precursors in an Orbitrap at 50,000 resolution with a normalized AGC set at 500 followed by maximum injection time set as “Auto” with a normalized collision energy setting of 65. Acquired MS/MS spectra were searched against a human Uniprot protein database along with a contaminant protein database, using a SEQUEST and percolator validator algorithms in the Proteome Discoverer 2.4 software (Thermo Scientific). The precursor ion tolerance was set at 10 ppm and the fragment ions tolerance was set at 0.02 Da along with methionine oxidation included as dynamic modification. Carbamidomethylation of cysteine residues and TMT16 plex (304.2071Da) was set as a static modification of lysine and the N-termini of the peptide. Trypsin was specified as the proteolytic enzyme, with up to 2 missed cleavage sites allowed. Searches used a reverse sequence decoy strategy to control for the false peptide discovery and identifications were validated using percolator software. Reporter ion intensities were adjusted to correct for the impurities according to the manufacturer’s specification and the abundances of the proteins were quantified using the summation of the reporter ions for all identified peptides. The reporter abundances were normalized across all the channels to account for equal peptide loading. Data from the first replicate of the DMSO, rapamycin, and temsirolimus conditions was excluded from the analysis due to their being identified as outliers in Principal Component Analysis. As such, mean abundance values for DMSO, rapamycin, and temsirolimus were calculated from two replicates while mean abundance values for ridaforolimus was calculated from three replicates.

## Supplementary Material

Supplement 1

1

## Significance

Due to a lack of effective antivirals available to combat SARS-CoV-2 infection and the disease it causes (COVID-19), existing drugs that are clinically-approved for use in humans are being considered for deployment during this protracted pandemic. Rapamycin is an immunosuppressant that is administered to humans suffering from cancer, autoimmunity, atherosclerosis, and organ transplant rejection. While generally recognized for its inhibitory effects on the adaptive immune response, we previously showed that rapamycin can also impair intrinsic immunity, the immune system present within all cells of the body. Here, we show that rapamycin and rapamycin analogs (rapalogs) promote the first step of the SARS-CoV-2 infection cycle—entry into cells—by inducing the destruction of antiviral proteins that stand guard during virus invasion.
