# Rapalogs downmodulate intrinsic immunity and promote cell entry of SARS-CoV-2

**DOI:** 10.1101/2021.04.15.440067

**Published:** 2021-12-10

**Authors:** Guoli Shi, Abhilash I. Chiramel, Saliha Majdoul, Kin Kui Lai, Tirhas Dempsey, Adam Kenney, Ashley Zani, Adrian Eddy, Lizhi Zhang, Sudipto Das, Paul A. Beare, Thorkell Andresson, Swagata Kar, Jacob S. Yount, Sonja M. Best, Alex A. Compton

**Affiliations:** 1 HIV Dynamics and Replication Program, Center for Cancer Research, National Cancer Institute, Frederick, MD, USA; 2 Laboratory of Virology, Rocky Mountain Laboratories, National Institute of Allergy and Infectious Diseases, Hamilton, MT, USA; 3 Department of Microbial Infection and Immunity, The Ohio State University, Columbus, OH, USA; 4 Protein Characterization Laboratory, Center for Cancer Research, National Cancer Institute, Frederick, MD, USA; 5 Laboratory of Bacteriology, Rocky Mountain Laboratories, National Institute of Allergy and Infectious Diseases, Hamilton, MT, USA; 6 Bioqual, Rockville, MD, USA

**Keywords:** rapamycin, rapalog, mTOR inhibitor, IFITM, interferon, SARS-CoV-2, COVID-19, coronavirus, membrane fusion, everolimus, temsirolimus, ridaforolimus, TFEB, autophagy

## Abstract

SARS-CoV-2 infection in immunocompromised individuals is associated with prolonged virus shedding and the evolution of viral variants. Rapamycin and its analogs (rapalogs, including everolimus, temsirolimus, and ridaforolimus) are FDA-approved as mTOR inhibitors in clinical settings such as cancer and autoimmunity. Rapalog use is commonly associated with increased susceptibility to infection, which has been traditionally explained by impaired adaptive immunity. Here, we show that exposure to rapalogs increases susceptibility to SARS-CoV-2 infection in tissue culture and in immunologically naïve rodents by antagonizing the cell-intrinsic immune response. By identifying one rapalog (ridaforolimus) lacking this function, we demonstrate that rapalogs promote Spike-mediated entry into cells by triggering the lysosomal degradation of IFITM2 and IFITM3. Rapalogs that promote virus entry inhibit the mTOR-mediated phosphorylation of TFEB, a transcription factor controlling lysosome biogenesis and degradative capacity. In the hamster model of infection, injection of rapamycin four hours prior to virus exposure resulted in elevated virus titers in lungs and accelerated weight loss, while ridaforolimus had milder effects. Furthermore, rapamycin significantly elevated mouse-adapted SARS-CoV-2 titers in lungs of mice. Overall, our findings indicate that preexisting use of certain rapalogs may elevate host susceptibility to SARS-CoV-2 infection and disease by activating a lysosome-mediated suppression of intrinsic immunity.

## Summary

Rapamycin is an immunosuppressant used in humans to treat cancer, autoimmunity, and other disease states. Here, we show that rapamycin and related compounds promote the first step of the SARS-CoV-2 infection cycle—entry into cells—by disarming cell-intrinsic immune defenses.

## Introduction

Severe acute respiratory syndrome (SARS) coronavirus (CoV)-2 emerged in humans in 2019 following a species jump from bats and a possible intermediate animal host and is the cause of COVID-19, a respiratory and multi-organ disease of variable severity (Wu et al., 2020; Zhu et al., 2020). The characterization of virus-host interactions that dictate SARS-CoV-2 infection and COVID-19 severity is a major priority for public health (Zhang et al., 2020a). Immune impairment, such as that resulting from cancer, has been associated with prolonged SARS-CoV-2 shedding and the seeding of “super-spreader” events (Avanzato et al., 2020; Aydillo et al., 2020; Baang et al., 2021; Tarhini et al., 2021; Wei et al., 2020).

**Figure 1:**
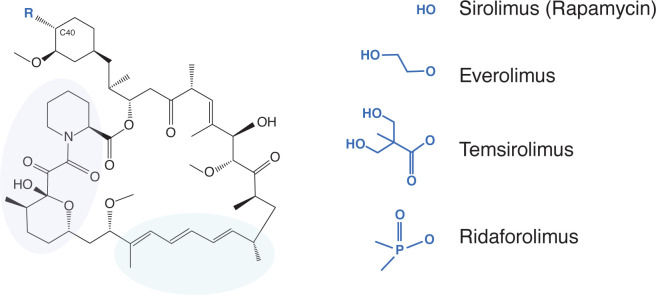
Rapamycin and its analogs share a macrolide structure but differ by the functional group present at carbon-40. Violet and green bubbles indicate the FKBP- and mTOR-binding sites, respectively.

One group of compounds being considered for the treatment of COVID-19-related immunopathology are rapamycin (sirolimus, Rapamune) and rapamycin analogs (rapalogs) (Appelberg et al., 2020; Blagosklonny, 2020; Ciliberto et al., 2020; Gordon et al., 2020; Husain and Byrareddy, 2020; Omarjee et al., 2020; Ramaiah, 2020; Terrazzano et al., 2020; Willyard, 2020; Zhavoronkov, 2020; Zheng et al., 2020; Zhou et al., 2020). As Food and Drug Administration-approved inhibitors of mammalian target of rapamycin (mTOR) kinase, these macrolide compounds are used therapeutically to inhibit the processes of cancer, autoimmunity, graft versus host disease, atherosclerosis, and aging (Laplante and Sabatini, 2012). Rapalogs, including everolimus (RAD-001), temsirolimus (Torisel, CCI-779), and ridaforolimus (deforolimus, AP-23573), were developed to decrease the half-life of rapamycin *in vivo* in order to minimize the systemic immunosuppression caused by rapamycin use, which is associated with increased susceptibility to infections (Abdel-Magid, 2019; Benjamin et al., 2011; Bertram et al., 1998; Lamming et al., 2013; Vignot et al., 2005). Differing by only a single functional group at carbon-40 (Figure 1), it is believed that rapamycin and rapalogs share the same molecular mechanism of action to inhibit mTOR kinase—they bind to FK506-binding proteins (FKBP) and the resulting complex physically interacts with mTOR and disrupts its signaling (Abdel-Magid, 2019; Marz et al., 2013).

Activation of mTOR promotes cell growth, cell proliferation, and cell survival (Zoncu et al., 2011). In addition, mTOR activation promotes pro-inflammatory T-cell differentiation and mTOR inhibitors have been used to block lymphocyte proliferation and cytokine storm (Chi, 2012). Since respiratory virus infections like SARS-CoV-2 can cause disease by provoking hyper-inflammatory immune responses that result in immunopathology (Mangalmurti and Hunter, 2020; Manjili et al., 2020; Mehta et al., 2020), rapalogs are being tested as treatments to decrease viral disease burden. At least three active clinical trials have been designed to test the impact of rapamycin on COVID-19 severity in infected patients (NCT04461340, NCT04341675, NCT04371640).

In addition to their potential utility for mitigating disease in individuals already infected by SARS-CoV-2, there are also calls to use rapalogs as antiviral agents to inhibit virus infection itself (i.e. as a prophylactic) (Bischof et al., 2021). It was recently shown that rapalogs inhibit SARS-CoV-2 replication when added to cells post-infection (Mullen et al., 2021), attesting to a potential use of rapalogs as antivirals in infected individuals. Furthermore, it was reported that mTOR activation promotes infection by the human coronavirus that causes Middle East Respiratory Syndrome (MERS-CoV) (Kindrachuk et al., 2015). Nonetheless, rapalogs are known to induce an immunosuppressed state in humans characterized by an increased rate of infections, including those caused by respiratory viruses. Furthermore, rapamycin administration concurrent with virus challenge has been shown to promote Influenza A replication in mice and to exacerbate viral disease (Alsuwaidi et al., 2017b; Huang et al., 2017), but the mechanism was unknown. We previously found that exposure of human and murine cells to rapamycin induced the lysosomal degradation of a select group of cellular proteins, including the interferon-inducible transmembrane (IFITM) proteins, and rendered cells more permissive to infection by Influenza A virus and gene-delivering lentiviral vectors (Ozog et al., 2019; Shi et al., 2018a). IFITM1, IFITM2, and IFITM3 are expressed constitutively in a variety of tissues, are further upregulated by type-I and type-II interferons, and are important components of cell-intrinsic immunity, the antiviral network that defends individual cells against virus invasion (Majdoul and Compton, 2021; Shi et al., 2017). Nonetheless, it remained to be determined how rapamycin-mediated regulation of intrinsic immunity impacts host susceptibility to virus infection *in vivo*.

In this report, we show that rapalogs differentially counteract the constitutive and interferon-induced antiviral state in lung cells and increase permissiveness to SARS-CoV-2 infection. We found that the enhancing effect of rapalogs on SARS-CoV-2 infection is functionally linked to their capacity to trigger degradation of IFITM proteins, particularly IFITM2 and IFITM3. By identifying a rapalog that lacks this activity, we found that IFITM protein turnover and SARS-CoV-2 infection enhancement are associated with activation of TFEB, a master regulator of lysosome function that is regulated by mTOR. Administration of rapamycin to naive rodents four hours prior to experimental SARS-CoV-2 infection increased virus replication and viral disease severity, indicating for the first time that suppression of intrinsic immunity by rapamycin contributes to its immunosuppressive properties *in vivo*.

## Results

### Select rapalogs promote SARS-CoV-2 infection and downmodulate IFITM proteins in lung cells

To assess how rapamycin and rapalogs impact SARS-CoV-2 infection, we took advantage of a pseudovirus system based on human immunodeficiency virus (HIV). This pseudovirus (HIV-CoV-2 S) is limited to a single round of infection, cell entry is mediated by SARS-CoV-2 Spike, and infection of target cells is measured by luciferase activity. SARS-CoV-2 can enter cells via multiple routes, and sequential proteolytic processing of Spike is essential to this process. SARS-CoV-2 Spike is cleaved at a polybasic motif (RRAR) located at the S1/S2 boundary by furin-like proteases in virus-producing cells prior to release. Subsequently, the S2’ site is cleaved by the trypsin-like proteases TMPRSS2 on the target cell surface or cathepsins B and L in target cell endosomes, triggering membrane fusion at those sites (Bestle et al., 2020; Hoffmann et al., 2020a; Hoffmann et al., 2020b).

**Figure 2:**
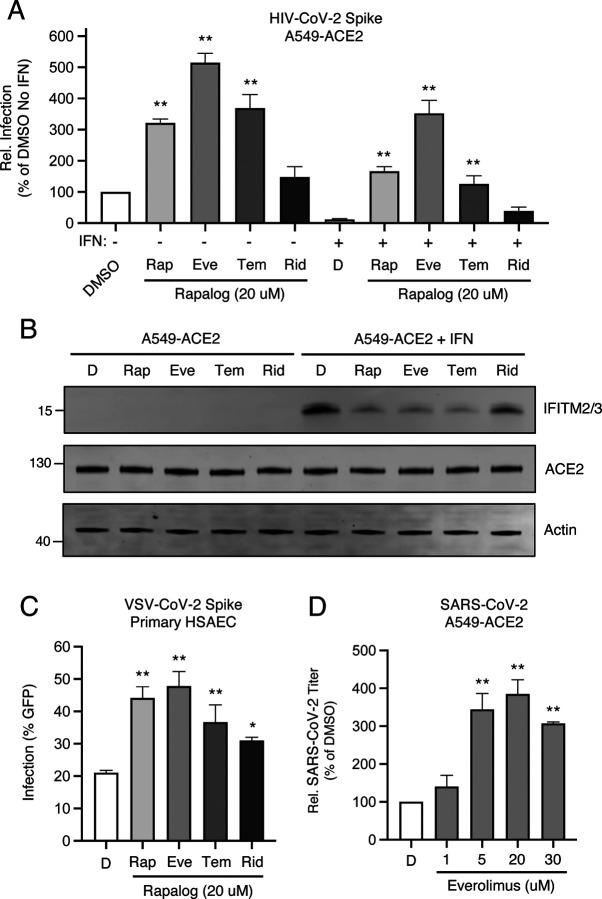
Rapalogs promote SARS-CoV-2 infection in lung epithelial cells to different extents by counteracting the intrinsic antiviral state (A) A549-ACE2 were treated with or without type I interferon (250 U/mL) for 18 hours and then treated with 20 μM rapamycin (Rap), everolimus (Eve), temsirolimus (Tem), ridaforolimus (Rid), or an equivalent volume of DMSO (D) for 4 hours. HIV-CoV-2 (100 ng p24 equivalent) was added to cells and infection was measured by luciferase activity at 48 hours post-infection. Luciferase units were normalized to 100 in the DMSO condition in the absence of interferon. (B) A549-ACE2 cells from (A) were subjected to SDS-PAGE and Western blot analysis. Immunoblotting was performed with anti-IFITM2/3, anti-ACE2, and anti-actin (in that order) on the same nitrocellulose membrane. Numbers and tick marks indicate size (kilodaltons) and position of protein standards in ladder. (C) Primary HSAEC were treated with 20 μM Rap, Eve, Tem, Rid, or an equivalent volume of DMSO for 4 hours. VSV-CoV-2 (50 μL) was added to cells and infection was measured by GFP expression at 24 hours post-infection using flow cytometry. (D) A549-ACE2 were treated with varying concentrations of Eve or DMSO for 4 hours. SARS-CoV-2 (nCoV-WA1–2020; MN985325.1) was added to cells at an MOI of 0.1 and infectious titers were measured in VeroE6 cells by calculating the TCID_50_ per mL of supernatants recovered at 24 hours post-infection. TCID_50_ per mL values were normalized to 100 in the DMSO condition. Means and standard error were calculated from 3–5 experiments. Statistical analysis was performed with one-way ANOVA and asterisks indicate significant difference from DMSO. *, p < 0.05; **, p < 0.01. Rel.; relative.

Using A549-ACE2 (transformed human lung epithelial cells that, in addition to overexpressing the ACE2 receptor, naturally express TMPRSS2), we pre-treated cells with 20 μM rapamycin, everolimus, temsirolimus, ridaforolimus, or DMSO (vehicle control) for four hours and then challenged cells with HIV-CoV-2. Interestingly, we found that rapalogs promoted Spike-mediated infection to different extents: rapamycin, everolimus, and temsirolimus significantly enhanced infection (up to 5-fold) while ridaforolimus did not (Figure 2A). To determine whether rapalogs promote cell permissiveness to infection by upregulating dependency factors or by downregulating restriction factors, we performed the same experiment in cells pre-treated with type-I interferon. While type-I interferon suppressed infection by approximately 90%, the addition of rapamycin, everolimus, and temsirolimus resulted in rescue of infection by up to 20-fold (Figure 2A). As a result, infection levels were partially restored to those achieved in the absence of interferon, with everolimus having the greatest boosting effect and ridaforolimus having no effect. Therefore, rapalogs differentially promote SARS-CoV-2 Spike-mediated infection by counteracting intrinsic antiviral defenses in lung cells to different extents.

Type-I interferon treatment of A549-ACE2 resulted in upregulation of *IFITM2* and *IFITM3*, as detected by an antibody recognizing both proteins in whole cell lysates (Figure 2B). A549-ACE2 cells express low but detectable levels of IFITM2/3 in the absence of interferon treatment (Supplemental Figure 1A). Consistent with our previous publication, addition of rapamycin resulted in substantial loss of IFITM2/3 protein levels from cells. In a manner that mirrored the differential effects of rapalogs on pseudovirus infection, everolimus and temsirolimus diminished IFITM2/3 levels to a similar extent as rapamycin while ridaforolimus reduced IFITM2/3 to a lesser extent (Figure 2B). In contrast, ACE2 levels were not affected by interferon nor by rapalog treatment. Therefore, rapamycin derivatives may facilitate infection by antagonizing constituents of intrinsic immunity, including IFITM2/3, and this activity is determined by the chemical moiety found at carbon-40 of the macrolide structure.

To extend our findings to primary lung cells, we performed similar experiments in human small airway epithelial cells (HSAEC). While these cells were not permissive to HIV-CoV-2, they were susceptible to infection by pseudovirus based on vesicular stomatitis virus (VSV-CoV-2) whereby infection is reported by GFP expression. Pre-treatment of HSAEC with rapalogs enhanced VSV-CoV-2 infection to varying extents, but as observed in A549-ACE2 cells, everolimus exhibited the greatest effect and ridaforolimus, the least. Endogenous IFITM3 was readily detected in HSAEC under basal conditions (in the absence of interferon), while IFITM1 was barely detected and IFITM2 was not detected at all, and IFITM3 levels were downmodulated differentially by rapalogs (Supplemental Figure 1B). siRNA-mediated knockdown of IFITM3 in HSAEC resulted in enhanced VSV-CoV-2 infection, indicating that IFITM3 restricts Spike-mediated infection in these cells (Supplemental Figure 1C). We also treated semi-transformed nasal epithelial cells known as UNCNNTS2 with rapalogs in order to assess an impact on endogenous IFITM3 levels. As observed in HSAEC, downmodulation of IFITM3 occurred following treatment of UNCNNTS2 with rapamycin, everolimus, temsirolimus, and to a lesser extent, ridaforolimus (Supplemental Figure 1D).

Since 20 μM quantities of rapalogs promoted pseudovirus infection mediated by SARS-CoV-2 Spike, we tested how pretreatment of A549-ACE2 cells with varying amounts of everolimus impacted infection by replication-competent SARS-CoV-2. We observed a dose-dependent enhancement of infectious SARS-CoV-2 yield in supernatants of infected cells, (up to 4-fold) (Figure 2D). Therefore, since everolimus boosts pseudovirus infection and SARS-CoV-2 infection to similar extents, and since Spike is the only viral component shared between the two sources of infection, Spike-mediated cellular entry is the infection stage inhibited by the intrinsic defenses that are sensitive to downmodulation by rapalogs.

### Rapalogs facilitate cell entry mediated by various viral fusion proteins

**Figure 3:**
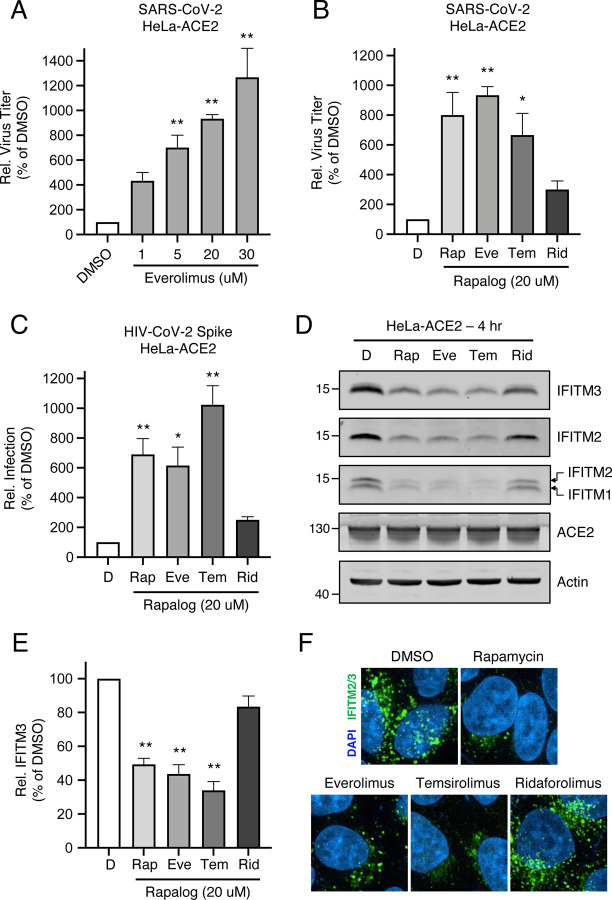
Rapalogs promote SARS-CoV-2 infection in HeLa-ACE2 cells. (A) HeLa-ACE2 were treated with varying concentrations of Eve or DMSO for 4 hours. SARS-CoV-2 (nCoV-WA1–2020; MN985325.1) was added to cells at an MOI of 0.1 and infectious titers were measured in VeroE6 cells by calculating the TCID_50_ of supernatants recovered at 24 hours post-infection. TCID_50_ per mL values were normalized to 100 in the DMSO condition. (B) HeLa-ACE2 were treated with 20 μM Rap, Eve, Tem, Rid, or an equivalent volume of DMSO for 4 hours. SARS-CoV-2 (nCoV-WA1–2020; MN985325.1) was added to cells at an MOI of 0.1 and infectious titers were measured in VeroE6 cells by calculating the TCID_50_ per mL of supernatants recovered at 24 hours post-infection. TCID_50_ per mL values were normalized to 100 in the DMSO condition. (C) HeLa-ACE2 were treated with 20 μM Rap, Eve, Tem, Rid, or an equivalent volume of DMSO for 4 hours. HIV-CoV-2 (100 ng p24 equivalent) was added to cells and infection was measured by luciferase activity at 48 hours post-infection. Luciferase units were normalized to 100 in the DMSO condition. (D) HeLa-ACE2 cells from (C) were subjected to SDS-PAGE and Western blot analysis. Immunoblotting was performed with anti-IFITM2, anti-IFITM1, anti-IFITM3, anti-ACE2, and anti-actin (in that order) on the same nitrocellulose membrane. (E) IFITM3 levels from (D) were normalized to actin levels and summarized from 5 independent experiments. (F) HeLa-ACE2 were treated with 20 μM Rap, Eve, Tem, Rid, or an equivalent volume of DMSO for 4 hours and cells were fixed, stained with DAPI and anti-IFITM2/3, and imaged by confocal immunofluorescence microscopy. Images represent stacks of 5 Z-slices and one representative image is shown per condition. Means and standard error were calculated from 3–5 experiments. Statistical analysis was performed with one-way ANOVA and asterisks indicate significant difference from DMSO. *, p < 0.05; **, p < 0.01. Rel.; relative.

In order to gain a greater mechanistic understanding of the effects of rapalogs on SARS-CoV-2 infection, we took advantage of HeLa cells overexpressing ACE2 (HeLa-ACE2). HeLa-ACE2 were pre-treated for four hours with increasing amounts of everolimus and then challenged with SARS-CoV-2. Everolimus increased titers of infectious virus released into supernatants in a dose-dependent manner, and to a greater extent than was observed for A549-ACE2 cells (Figure 3A). Furthermore, we found that pre-treatment of cells with 20 μM amounts of rapalogs enhanced SARS-CoV-2 titers to varying extents (Figure 3B). Rapamycin, everolimus, and temsirolimus significantly boosted SARS-CoV-2 infection (up to 10-fold) while ridaforolimus had less of an impact. We also performed infections of HeLa-ACE2 with HIV-CoV-2 pseudovirus, and the results were similar—ridaforolimus was inactive while the other three compounds significantly boosted Spike-mediated infection (Figure 3C). To test the link between infection enhancement and downmodulation of IFITM proteins by rapalogs, we probed for levels of IFITM3, IFITM2, and IFITM1 by immunoblotting whole cell lysates and by immunofluorescence in intact cells using specific antibodies. All IFITM proteins were readily detected in HeLa-ACE2 in the absence of interferon. IFITM3, IFITM2, and IFITM1 were significantly downmodulated following treatment with rapamycin, everolimus, and temsirolimus, but ridaforolimus had little to no effect on their expression (Figure 3D–E). In contrast, ridaforolimus reduced levels of IFITM proteins to a lesser extent. Furthermore, prolonged treatment (24 hours) of cells with everolimus and temsirolimus resulted in strong suppression of IFITM2 and IFITM3 protein levels (Supplemental Figure 2A). We confirmed that depletion of IFITM proteins by rapalogs occurs at the post-translational level, since bafilomycin A1, an inhibitor of endolysosomal acidification and function, prevented their loss (Supplemental Figure 2B). We previously showed that lysosomal degradation of IFITM3 triggered by rapamycin is maintained in *ATG9A* knockout cells lacking functional autophagosomes (Shi et al., 2018b). Here, we used SAR405, a selective inhibitor of phosphatidylinositol-3-phosphate (PI3P) production by vps34/PI3KC3 (Ronan et al., 2014) to prevent macroautophagy induction. At a concentration sufficient to reduce intracellular PI3P (Supplemental Figure 2C), SAR405 did not prevent degradation of IFITM2/3 following treatment with rapamycin, everolimus, or temsirolimus (Supplemental Figure 2D). Therefore, rapamycin and specific rapalogs trigger the degradation of endogenous factors mediating intrinsic resistance to SARS-CoV-2 infection, including the IFITM proteins, by promoting their turnover in lysosomes via a selective autophagy pathway.

**Figure 4:**
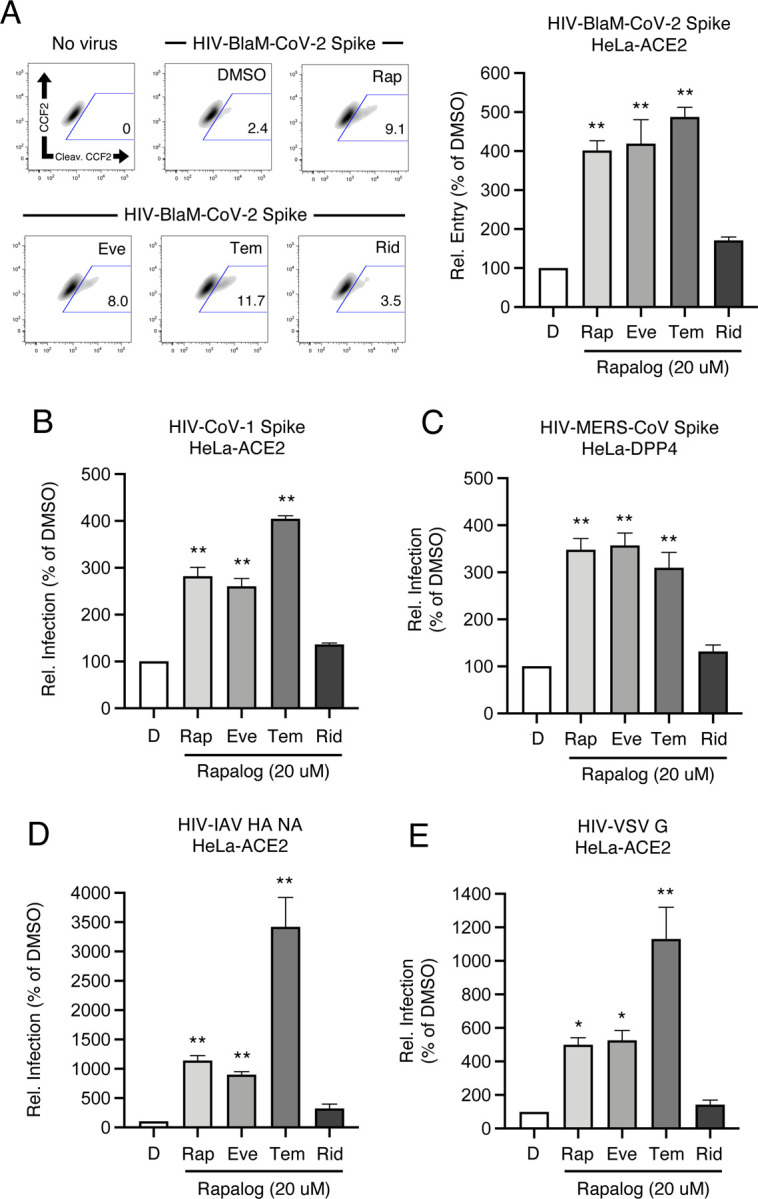
Rapalogs promote cell entry mediated by diverse viral fusion proteins. (A) HeLa-ACE2 were treated with 20 μM Rap, Eve, Tem, Rid, or an equivalent volume of DMSO for 4 hours. HIV-CoV-2 S pseudovirus incorporating BlaM-Vpr (HIV-BlaM-CoV-2) was added to cells for 2 hours and washed. Cells were incubated with CCF2-AM for an additional 2 hours and fixed. Cleaved CCF2 was measured by flow cytometry. Dot plots visualized as density plots from one representative experiment are shown on the left and the percentage of CCF2+ cells which exhibit CCF2 cleavage is indicated. Summary data representing the average of four experiments is shown on the right. (B) HIV-CoV-1, (C) HIV-MERS-CoV, (D) HIV-IAV HA, or (E) HIV-VSV G were added to HeLa-ACE2 or HeLa-DPP4 cells as in (A) and infection was measured by luciferase activity at 48 hours post-infection. Luciferase units were normalized to 100 in the DMSO condition. Means and standard error were calculated from 3–4 experiments. Statistical analysis was performed with one-way ANOVA and asterisks indicate significant difference from DMSO. *, p < 0.05; **, p < 0.01. Rel.; relative.

Enveloped virus entry into cells is a concerted process involving virus attachment to the cell surface followed by fusion of cellular and viral membranes. Since IFITM proteins are known to inhibit virus-cell membrane fusion, we quantified the terminal stage of HIV-CoV-2 entry by tracking the cytosolic delivery of beta-lactamase (BlaM) in single cells. We found that treatment of cells with rapamycin, everolimus, and temsirolimus resulted in enhanced HIV-CoV-2 entry while ridaforolimus had no such effect (Figure 4A). To measure whether rapalogs promote the cell entry process driven by other coronavirus Spike proteins, we produced HIV incorporating Spike from SARS-CoV (HIV-CoV-1) or MERS-CoV (HIV-MERS-CoV). Infections by both HIV-CoV-1 and HIV-MERS-CoV were elevated by rapalog treatment in HeLa-ACE2 and HeLa-DPP4, respectively, although the extent of enhancement was lower than that observed with HIV-CoV-2 (Figure 4B–C). Consistently, ridaforolimus was the least active among the rapalogs tested and it did not significantly promote pseudovirus infection. Since we previously showed that rapamycin promoted the cellular entry of Influenza A virus and VSV-G pseudotyped lentiviral vectors (Shi et al., 2018b), we also assessed infection of pseudoviruses incorporating hemagglutinin (HIV-HA) or VSV G (HIV-VSV G). Rapamycin, everolimus, and especially temsirolimus boosted HA- and VSV G-mediated infections (up to 30-fold and 11-fold, respectively), while ridaforolimus was inactive (Figure 4D–E). Since IFITM proteins have been previously shown to inhibit infection by SARS-CoV-1, MERS-CoV, VSV, and Influenza A virus (Majdoul and Compton, 2021), these data suggest that rapalogs promote infection, at least in part, by lowering the barrier to virus entry imposed by IFITM proteins.

### IFITM2/3 mediate the rapalog-sensitive barrier to SARS-CoV-2 infection in HeLa-ACE2

**Figure 5:**
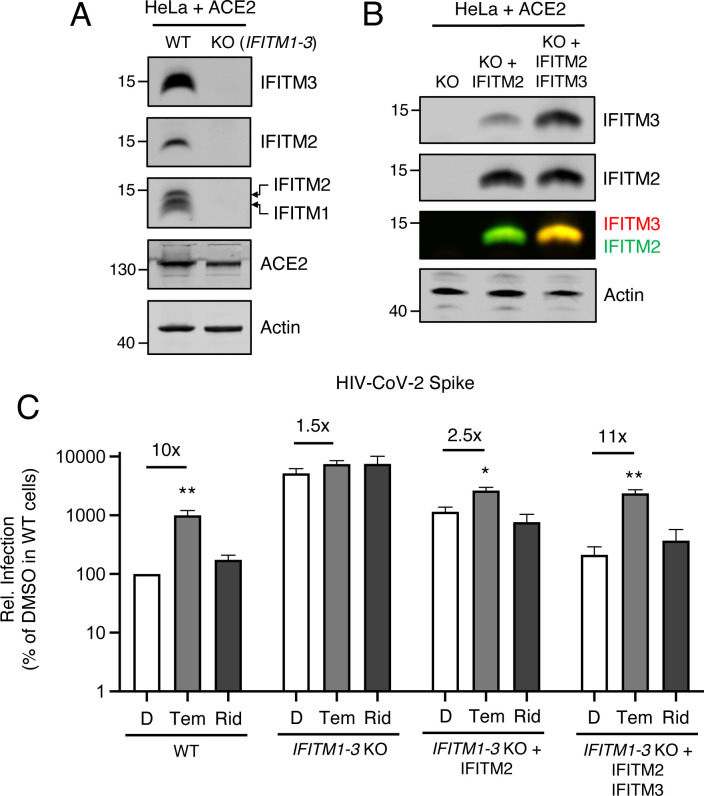
Downmodulation of IFITM2 and IFITM3 causes rapalog-specific enhancement of Spike-mediated entry in HeLa-ACE2. (A) HeLa WT and HeLa *IFITM1–3* KO cells were transiently transfected with ACE2 for 24 hours. Whole cell lysates were subjected to SDS-PAGE and Western blot analysis. Immunoblotting was performed with anti-IFITM2, anti-IFITM3, anti-IFITM1, anti-ACE2, and anti-actin (in that order) on the same nitrocellulose membrane. (B) HeLa *IFITM1–3* KO were transfected with IFITM2 or IFITM2 and IFITM3 and SDS-PAGE and Western blot analysis was performed. (C) HIV-CoV-2 was added to transfected cells from (B) and infection was measured by luciferase activity at 48 hours post-infection. Luciferase units were normalized to 100 in HeLa WT cells treated with DMSO. Means and standard error were calculated from 5 experiments. Statistical analysis was performed with one-way ANOVA and asterisks indicate significant difference from nearest DMSO condition. *, p < 0.05; **, p < 0.01. Rel.; relative.

To formally address how rapalog-mediated depletion of IFITM proteins impacts SARS-CoV-2 Spike-mediated entry, we used HeLa cells in which IFITM1, IFITM2, and IFITM3 are knocked out (*IFITM1–3* KO) and introduced human ACE2 by transient transfection (Figure 5A). IFITM2 alone or both IFITM2 and IFITM3 were restored in *IFITM1–3* KO cells by transient overexpression (Figure 5B) and cells were challenged with HIV-CoV-2. Relative to WT cells, HIV-CoV-2 infection was approximately 50-fold higher in *IFITM1–3* KO cells, indicating that endogenous IFITM proteins restrict SARS-CoV-2 Spike-mediated infection. Furthermore, while temsirolimus significantly promoted infection by 10-fold in WT cells, little to no enhancement was observed in *IFITM1–3* KO cells (Figure 5C). Ectopic expression of IFITM2 inhibited infection and partially restored sensitivity to temsirolimus, while the combination of IFITM2 and IFITM3 restricted infection further and fully restored temsirolimus sensitivity. These findings indicate that temsirolimus promotes Spike-mediated infection in HeLa-ACE2 cells by lowering levels of endogenous IFITM2 and IFITM3.

### Rapalogs differentially activate a lysosomal degradation pathway orchestrated by TFEB

**Figure 6:**
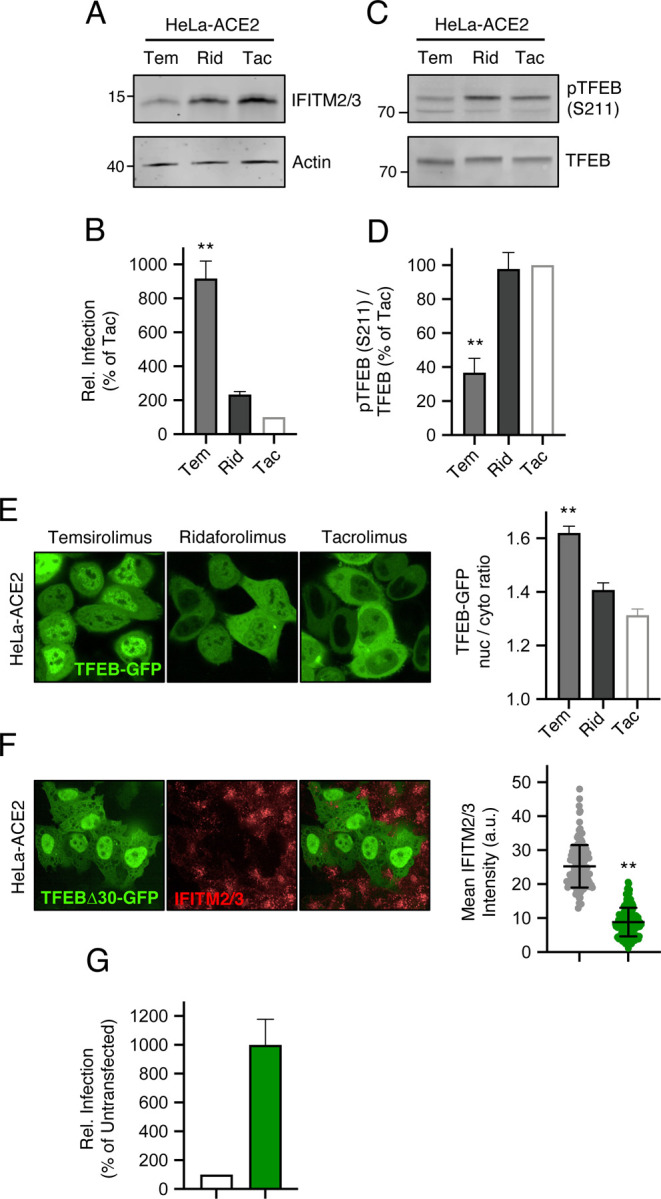
Enhancement of infection by select rapalogs is associated with inhibition of mTOR-mediated phosphorylation of TFEB. (A) HeLa-ACE2 were treated with 20 μM Tem, Rid, or tacrolimus (Tac) for 4 hours and whole cell lysates were subjected to SDS-PAGE and Western blot analysis. Immunoblotting was performed with anti-IFITM2/3 and anti-actin. (B) HIV-CoV-2 was added to HeLa-ACE2 treated as in (A) and infection was measured by luciferase activity at 48 hours post-infection. Luciferase units were normalized to 100 in the Tac condition. (C) HeLa-ACE2 were treated with 20 μM Tem, Rid, or Tac for 4 hours and whole cell lysates were subjected to SDS-PAGE and Western blot analysis. Immunoblotting was performed with anti-TFEB and anti-pTFEB (S211). (D) pTFEB (S211) levels were divided by total TFEB levels and summarized as an average of 3 experiments. (E) HeLa-ACE2 were transfected with TFEB-GFP for 24 hours, treated with Tem, Rid or Tac for 4 hours, stained with DAPI and CellMask, and imaged by high-content confocal fluorescence microscopy. Representative images from each condition are shown on the left. The ratio of nuclear to cytoplasmic TFEB-GFP was calculated in individual cells and the average ratio derived from approximately 200–300 cells per condition is shown to the right. (F) HeLa-ACE2 were transfected with 0.5 μg TFEBΔ30-GFP for 24 hours, fixed, stained with anti-IFITM2/3, and imaged by confocal immunofluorescence microscopy. The relative intensity of IFITM2/3 levels was compared between approximately 150 GFP-negative and 150 GFP-positive cells. (G) HeLa-ACE2 were transfected with 0.5 μg TFEBΔ30-GFP for 24 hours and HIV-CoV-2 (100 ng p24 equivalent) was added to cells. Infection was measured by luciferase activity at 48 hours post-infection. Luciferase units were normalized to 100 in the non-transfected condition. Means and standard error were calculated from 3 experiments, except for TFEB-GFP imaging experiments, for which 2 experiments (transfections) were performed. Statistical analysis was performed with one-way ANOVA and asterisks indicate significant difference from Tac. *, p < 0.05; **, p < 0.01. Rel.; relative.

Since rapamycin and rapalogs are known to inhibit signaling by mTOR by binding both mTOR and FKBP12 (and other FKBP members), we sought to determine whether mTOR binding and its inhibition are required for rapalog-mediated enhancement of SARS-CoV-2 infection. To that end, we tested the effect of tacrolimus (also known as FK506), a macrolide immunosuppressant that is chemically related to rapalogs but does not bind nor inhibit mTOR. Instead, tacrolimus forms a ternary complex with FKBP12 and calcineurin to inhibit the signaling properties of the latter (Kolos et al., 2018). In HeLa-ACE2 cells, a four-hour treatment of 20 μM tacrolimus did not reduce levels of IFITM2/3 (Figure 6A), nor did it boost HIV-CoV-2 infection (Figure 6B), while an equivalent amount of temsirolimus did both. These results suggest that FKBP12 binding is not sufficient for drug-mediated enhancement of SARS-CoV-2 infection. They also suggest that the extent to which mTOR is inhibited may explain the differential degree to which infection is impacted by the immunosuppressants examined in this study. Therefore, we surveyed the phosphorylation status of TFEB, a transcription factor that controls lysosome biogenesis and degradative processes carried out by lysosomes, such as autophagy (Roczniak-Ferguson et al., 2012). mTOR phosphorylates TFEB at serine 211 (S211), which promotes its sequestration in the cell cytoplasm and decreases its translocation into the nucleus (Martina et al., 2012; Roczniak-Ferguson et al., 2012; Settembre et al., 2012). Furthermore, this phosphorylation event was previously shown to be sensitive to inhibition by rapamycin and temsirolimus (Martina et al., 2012; Zhang et al., 2019). Using tacrolimus as a negative control, we found that temsirolimus significantly reduced S211 phosphorylation of endogenous TFEB in HeLa-ACE2 while ridaforolimus did not (Figure 6C–D). We also found that rapamycin, everolimus, and temsirolimus, but not ridaforolimus, inhibited TFEB S211 phosphorylation in A549-ACE2 cells (Supplemental Figure 3A–B). Loss of pTFEB (S211) following treatment with temsirolimus was associated with significantly elevated protein levels of several TFEB-regulated genes (cathepsin L, VAMP8, syntaxin 8, M6PR, and SCAMP2 (Di Fruscio et al., 2015; Palmieri et al., 2011)) as measured by mass spectrometry (Supplemental Figure 3C and Supplemental File 1). We assessed the subcellular localization of TFEB-GFP in HeLa-ACE2 treated with different compounds and found that, compared to ridaforolimus, temsirolimus induced a significantly greater accumulation of TFEB-GFP in the nucleus (Figure 6E). These findings may suggest that ridaforolimus exhibits a less potent inhibition of mTOR-mediated TFEB phosphorylation at S211. Therefore, nuclear translocation of TFEB is associated with IFITM2/3 degradation and increased cellular susceptibility to SARS-CoV-2 Spike-mediated infection. Consistent with a direct relationship between TFEB activation, IFITM2/3 turnover, and Spike-mediated cell entry, we found that ectopic expression of a constitutively active form of TFEB lacking the first 30 amino-terminal residues (Roczniak-Ferguson et al., 2012) was sufficient to trigger IFITM2/3 loss from cells (Figure 6F) and sufficient to increase susceptibility to HIV-CoV-2 infection (Figure 6G). In summary, our results employing functionally divergent rapalogs reveal a previously unrecognized immunoregulatory role played by the mTOR-TFEB-lysosome axis that affects the cell entry of SARS-CoV-2 and other viruses.

### Rapamycin enhances SARS-CoV-2 infection and viral disease *in vivo*

**Figure 7:**
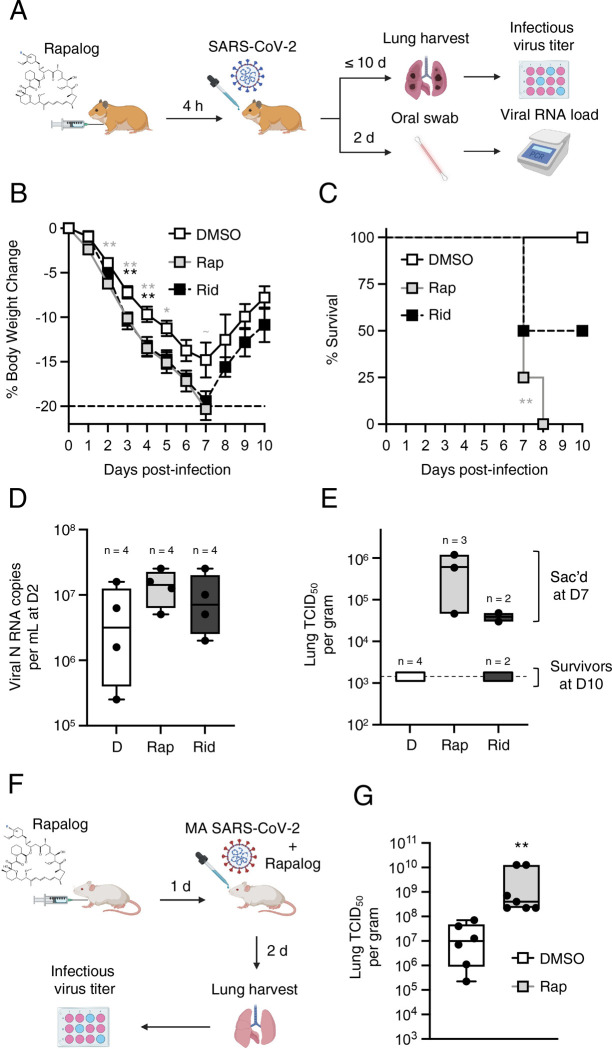
Rapamycin injection in hamsters prior to challenge with SARS-CoV-2 intensifies viral disease. (A) Schematic of intraperitoneal injections and intranasal SARS-CoV-2 challenge in hamsters. Golden Syrian hamsters were injected with 3 mg/kg Rap, Rid, or equivalent amounts of DMSO (4 animals per group). Four hours later, hamsters were infected intranasally with 6 × 10^3^ plaque forming units of SARS-CoV-2. At 2 days post-infection, oral swabs were taken and used for measurement of oral viral RNA load by qPCR. At 10 days post-infection (or earlier, if more than 20% of weight loss occurred), hamsters were euthanized, and lungs were harvested for determination of infectious virus titer by TCID_50_ assay in Vero-TMPRSS2 cells (B) Mean body weight and standard error for each treatment group is plotted by day post-infection. (C) Kaplan-Meier survival curves were generated according to the dates of euthanasia (or in one case, when an animal was found dead). All 4 hamsters in the DMSO condition survived until the end of the study (10 days post-infection), while 2 hamsters in the Rid condition survived and none of the hamsters in the Rap condition survived. (D) Viral RNA copy number was determined by qPCR from material obtained by oral swab at 2 days post-infection. Data is depicted as box and whiskers plots. (E) Infectious virus titers in lungs were determined by TCID_50_ in Vero-TMPRSS2 cells. Data is depicted as floating bars and is grouped by brackets according to hamsters that survived until 10 days post-infection and those that were euthanized at 7 days post-infection. Statistical analysis in (B) was performed by student’s T test and asterisks indicate significant difference from DMSO (gray asterisks for Rap and black asterisks for Rid). Statistical analysis in (C) was performed by comparing survival curves between Rap and DMSO or Rid and DMSO using the Log-rank (Mantel-Cox) test. Gray asterisks indicate that the survival curve of Rap-treated hamsters differed significantly from the survival curve of DMSO-treated hamsters. (F) Schematic of intraperitoneal injections and intranasal mouse-adapted (MA) SARS-CoV-2 challenge in mice. C57BL/6 mice were injected with 3 mg/kg of Rap or an equivalent amount of DMSO (6 or 7 mice per group, respectively). The following day, mice were infected intranasally with 6 × 10^4^ TCID_50_ MA SARS-CoV-2. Mice received second and third injections of Rap or DMSO on the day of infection and day one post-infection, respectively. (G) Lungs were harvested from infected mice upon euthanasia at day 2 post-infection and infectious viral loads from lung homogenates were determined by TCID_50_ in Vero-TMPRSS2 cells. Geometric mean TCID_50_ per gram was calculated per treatment group and data is depicted as box and whiskers plots. Statistical analysis was performed with Mann-Whitney test and asterisks indicate significant difference from DMSO. *, p < 0.05; **, p < 0.01. Illustration created with BioRender.com.

Our findings from SARS-CoV-2 and pseudovirus infection of human cells demonstrate that rapamycin, everolimus, and temsirolimus can suppress intrinsic immunity at the post-translational level, while ridaforolimus does not. However, whether these compounds are functionally divergent when administered *in vivo* was unclear. Since temsirolimus is a prodrug of rapamycin (it is metabolized to rapamycin), and since rapamycin was previously shown to promote morbidity of Influenza A infection in mice (Alsuwaidi et al., 2017a; Huang et al., 2017), we tested how intraperitoneal injection of rapamycin, ridaforolimus, or DMSO before intranasal challenge with SARS-CoV-2 impacted virus replication and disease course in naïve hamsters (Figure 7A). Hamsters are a permissive model for SARS-CoV-2 because hamster ACE2 is sufficiently similar to human ACE2 to support productive infection. Furthermore, in contrast to transgenic mice expressing human ACE2 or mice infected with mouse-adapted (MA) SARS-CoV-2, hamsters exhibit severe disease characterized by lung pathology when high viral loads are achieved (Tostanoski et al., 2020). Four hamsters were randomly allocated to each group. Relative to DMSO treatment, hamsters injected with rapamycin four hours prior to challenge exhibited a significantly greater loss of body weight at days 2–5 post-infection (Figure 7B). In contrast, ridaforolimus treatment impacted weight loss to a slightly lesser extent, with significant differences from DMSO apparent at days 3 and 4 post-infection. While none of the hamsters treated with DMSO exhibited severe weight loss necessitating euthanasia and body weights recovered, three of the four animals treated with rapamycin were euthanized on day 7 post-infection, and the fourth animal was found dead on day 8. Meanwhile, two of the four hamsters treated with ridaforolimus met requirements for euthanasia on day 7 post-infection, and the two survivors recovered body weight between days 8 and 10 post-infection (Figure 7B). As a result, hamsters treated with rapamycin exhibited significantly reduced survival compared to the DMSO group (Figure 7C). Early SARS-CoV-2 replication was measured by quantitative PCR from oral swabs taken on day 2 post-infection. While there was large variance among hamsters in the DMSO group, rapamycin-treated hamsters exhibited a higher viral burden on average while ridaforolimus-treated hamsters exhibited more moderate increases (differences were not statistically significant) (Figure 7D). Terminal viral titers in lungs were also measured following euthanasia. Infectious virus was undetectable in the lungs of DMSO-treated hamsters on day 10 post-infection, indicative of viral clearance (Figure 7E). In contrast, infectious virus was detected in lungs of the three rapamycin-treated hamsters that were euthanized on day 7 post-infection (the fourth hamster that was found dead on day 8 was not examined). By comparison, virus detected in lungs of two ridaforolimus-treated hamsters euthanized on the same day were lower on average (differences were not statistically significant). No infectious virus was detected in lungs from the remaining two hamsters treated with ridaforolimus that survived until the end of the study. Overall, these results suggest that rapamycin administration just prior to SARS-CoV-2 exposure increases host susceptibility to infection and significantly increases morbidity and mortality in a manner that is associated with elevated virus replication.

We previously found that, like its human counterpart, murine IFITM3 is sensitive to depletion by rapamycin (Shi et al., 2018b). To confirm that rapamycin promotes host susceptibility to SARS-CoV-2 infection by modulating intrinsic resistance to infection, we injected C57BL/6 mice with rapamycin or DMSO four hours prior to challenge with MA SARS-CoV-2 and measured infectious viral burden in lungs upon euthanasia on day 2 post-infection (Figure 7F). We found that virus titers were significantly increased (144-fold) in rapamycin-treated mice compared to DMSO-treated mice (Figure 7G), suggesting that rapamycin downmodulates intrinsic barriers to infection *in vivo*.

## Discussion

By assessing their impact on infection at the single-cell and whole-organism level, we draw attention to an immunosuppressive property of rapamycin and some rapalogs that acts on cell-intrinsic immunity and increases cellular susceptibility to infection by SARS-CoV-2 and likely other pathogenic viruses. Side effects of rapalog use in humans, including increased risk of respiratory tract infections, are regularly attributed to immunosuppression of adaptive immunity (Sasongko et al., 2015). Indeed, rapalogs have been used to mitigate systemic immunopathology caused by T-cell responses, and this is one reason why they are being tested for therapeutic benefit in COVID-19 patients. However, by injecting rapamycin into immunologically naïve hosts just prior to virus challenge, it is unlikely that rapalogs used in our experiments modulated adaptive immunity against SARS-CoV-2. Therefore, while immunomodulation of adaptive immunity by rapalogs may provide benefit for patients already suffering from COVID-19, preexisting rapalog use may enhance susceptibility by counteracting cell-intrinsic immunity. The injection dose of rapamycin or ridaforolimus (3 mg/kg) that we administered once to hamsters or daily to mice, when adjusted for body surface area and an average human weight of 60 kg (Reagan-Shaw et al., 2008), equates to approximately 15 mg per human. This figure is similar to those administered to humans in clinical settings, such as the use of rapamycin for the treatment of glioblastoma (up to 10 mg daily for multiple days) or the use of temsirolimus for the treatment of renal cell carcinoma (25 mg once weekly) (Cloughesy et al., 2008; Sun et al., 2012; Vignot et al., 2005). Therefore, our results may provide new insight into how rapamycin and rapalogs elicit unintended immunocompromised states in humans.

**Figure 8:**
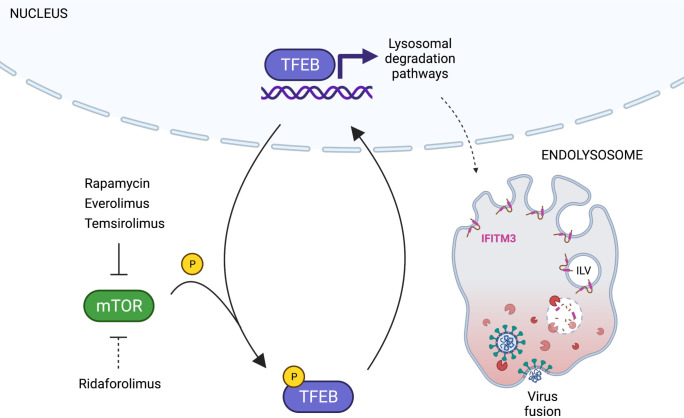
Model for rapalog-mediated enhancement of SARS-CoV-2 infection. Rapamycin and rapalogs everolimus and temsirolimus potently inhibit the phosphorylation of TFEB by mTOR, while ridaforolimus does not. As a result, TFEB translocates into the nucleus and induces genes functioning in lysosomal activities, including autophagy-related pathways. Nuclear TFEB triggers a microautophagy pathway that results in accelerated degradation of endolysosomal membrane proteins IFITM2 and IFITM3. Loss of IFITM2/3 promotes SARS-CoV-2 entry into cells by facilitating fusion between cellular and viral membranes.

By leveraging the differential functional properties of rapalogs, we reveal how the mTOR-TFEB-lysosome axis that impacts intrinsic resistance to SARS-CoV-2 and other virus infections. Specifically, rapamycin and select rapalogs (everolimus and temsirolimus) promote infection at the stage of cell entry, and this is functionally associated with nuclear accumulation of TFEB and the lysosomal degradation of IFITM proteins (Figure 8). While mTOR phosphorylates TFEB at S211 to promote the sequestration of TFEB in the cytoplasm, the phosphatase calcineurin dephosphorylates TFEB at this position to promote nuclear translocation (Medina et al., 2015). Therefore, the extent to which different rapalogs promote nuclear TFEB accumulation may be a consequence of differential mTOR inhibition and/or differential calcineurin activation. Calcineurin is activated by calcium release through the lysosomal calcium channel TRPML1 (also known as mucolipin-1) (Medina et al., 2015), and interestingly, it was shown that rapamycin and temsirolimus, but not ridaforolimus, promote calcium release by TRPML1 (Zhang et al., 2019). Therefore, it is worth examining whether TRPML1 or related lysosomal calcium channels are required for the effects of rapalogs on virus infection.

Lysosomes support the beginning (cellular entry) and end (cellular egress) of the SARS-CoV-2 infection cycle (Ghosh et al., 2020). Therefore, TFEB activation and its broad impacts on lysosome biogenesis and function could impact infection in multiple ways. It remains to be determined which TFEB target genes are, upon induction following rapalog treatment, directly responsible for increasing cellular susceptibility to infection. Possible candidates include cathepsins B and L, which are important host dependency factors for SARS-CoV-2 entry into cells lacking TMPRSS2 (Hoffmann et al., 2020b). In agreement with our finding that rapalogs differentially inhibit TFEB phosphorylation and promote its nuclear translocation, we show that temsirolimus, but not ridaforolimus, induced cathepsin L1 and other proteins encoded by TFEB-regulated genes. However, we show that rapalogs promote cell entry mediated by multiple viral fusion proteins, including Influenza A HA and vesicular stomatitis virus G, which do not utilize cathepsins B and L during their entry process (Schornberg et al., 2006). Together with our finding that enforced nuclear translocation of TFEB drives loss of IFITM2/3 from cells and concomitant enhancement of SARS-CoV-2 Spike-mediated infection, the mechanism by which select rapalogs promote virus infection likely involves the downmodulation of IFITM proteins, which broadly inhibit the entry of enveloped viruses in endosomes and at the plasma membrane (Chesarino et al., 2017; Desai et al., 2014; Guo et al., 2021; Li et al., 2013; Lin et al., 2013; Rahman et al., 2020). Overall, our findings reveal a previously unrecognized mechanism by which TFEB promotes virus infections—inhibition of cell-intrinsic defenses restricting virus entry. By extension, this work identifies TFEB as a therapeutic target, and inhibitors that limit levels of nuclear TFEB (such as calcineurin inhibitors) should be tested for broad-spectrum antiviral activity.

We previously demonstrated that treatment of cells with micromolar quantities of rapamycin induced the lysosomal degradation of IFITM2/3 via a pathway that is independent of macroautophagy yet dependent upon endosomal complexes required for transport (ESCRT)-mediated sorting of IFITM2/3 into intraluminal vesicles of late endosomes (Shi et al., 2018a). This degradation pathway resembles a non-canonical form of autophagy known as microautophagy, which occurs directly on endosomal or lysosomal membranes and involves membrane invagination (Schuck, 2020). Microautophagy in yeast and mammalian cells is characterized by ESCRT-dependent sorting of endolysosomal membrane proteins into intraluminal vesicles followed by their degradation by lysosomal hydrolases (Oku and Sakai, 2018)(Oku et al). During the process of endolysosomal membrane invagination during microautophagy, cytosolic proteins can also be internalized into intraluminal vesicles and degraded (Santambrogio and Cuervo, 2011; Tekirdag and Cuervo, 2018). Furthermore, microautophagy is known to be regulated by mTOR (Mijaljica et al., 2011; Sato et al., 2019), and mTOR inhibition triggers a ubiquitin- and ESCRT-dependent turnover of vacuolar (lysosomal) membrane proteins in yeast (Li et al., 2015; Yang et al., 2020). Overall, our findings suggest that select rapalogs induce a rapid endolysosomal membrane remodeling program controlled by TFEB, and IFITM proteins are among the client proteins subjected to this pathway. The full cast of cellular factors that orchestrate this selective degradation program in mammalian cells and the other client proteins subjected to it will need to be worked out. Interestingly, the E3 ubiquitin ligase NEDD4 was previously shown to ubiquitinate IFITM2 and IFITM3 and to induce their lysosomal degradation in mammalian cells (Chesarino et al., 2015; Compton et al., 2016), while Rsp5, the yeast ortholog of NEDD4, was shown to ubiquitinate vacuolar proteins turned over by microautophagy in yeast (Yang et al., 2019). Therefore, rapamycin and select rapalogs may upregulate NEDD4 function, resulting in selective degradation of a subset of the cellular proteome that includes IFITM proteins. Indeed, NEDD4 and the related NEDD4L are among the known target genes regulated by TFEB (Di Fruscio et al., 2015).

IFITM2 and IFITM3 are cell surface proteins which are targeted to endolysosomes via a tyrosine-based endocytic motif (YxxL) in the amino terminus, and phosphorylation regulates this process (Chesarino et al., 2014a; Chesarino et al., 2014b; Compton et al., 2016; Jia et al., 2012; Jia et al., 2014). While it lacks the YxxL motif present in IFITM2 and IFITM3, the carboxy-terminus of IFITM1 contains its own sorting signal and is responsible for a proportion of IFITM1 being localized to endolysosomes (Jia et al., 2015; Li et al., 2014). In the current study, we found that endogenous IFITM1, IFITM2, and IFITM3 are degraded following treatment with select rapalogs. This likely reflects a partial overlap in endolysosomal localization between IFITM1, IFITM2, and IFITM3. However, the relationship between IFITM proteins and human coronaviruses is complex. It was previously shown that IFITM3 facilitates replication of the seasonal coronavirus hCoV-OC43 (Zhao et al., 2014), while we and others recently showed that SARS-CoV-1 and SARS-CoV-2 infection is inhibited by ectopic and endogenous IFITM1, IFITM2, and IFITM3 from mice and humans (Huang et al., 2011; Shi et al., 2020; Winstone et al., 2021; Zang et al., 2020; Zhao et al., 2020). Intriguingly, mutants of human IFITM3 that lack the capacity to internalize into endosomes lost antiviral activity and promoted SARS-CoV-2 and MERS-CoV infection, revealing that IFITM3 can either inhibit or enhance infection depending on its subcellular localization (Shi et al., 2020; Zhao et al., 2018). Furthermore, one study reported that endogenous human IFITM proteins promoted infection by SARS-CoV-2 in certain human tissues (Bozzo et al., 2020). Overall, the net effect of human IFITM proteins on SARS-CoV-2 infection *in vivo* remains unclear. However, the impact of rapamycin in our experimental SARS-CoV-2 infections of hamsters and mice suggests that rapamycin-mediated loss of IFITM proteins favors virus infection and viral disease, consistent with IFITM proteins performing antiviral roles against SARS-CoV-2 in those species.

Other lines of evidence support an antiviral role for IFITM proteins during SARS-CoV-2 infection in humans. While SARS-CoV-2 infection has been shown to cause deficiencies in interferon synthesis and interferon response pathways, administration of type I interferon *in vivo* promotes SARS-CoV-2 clearance in hamsters and humans (Beyer and Forero, 2021). Notably, IFITM3 is among the most highly induced genes in primary human lung epithelial cells exposed to SARS-CoV-2 (Blanco-Melo et al., 2020; Hachim et al., 2020), and humans experiencing mild or moderative COVID-19 showed elevated induction of antiviral genes, including *IFITM1* and *IFITM3*, in airway epithelium compared to individuals suffering from more severe COVID-19 (Ziegler et al., 2021). Single nucleotide polymorphisms in human *IFITM3* known as ns12252 and rs34481144, which lead to IFITM3 loss-of-function, have been associated with severe outcomes following Influenza A virus infection as well as severe COVID-19 (Nikoloudis et al., 2020; Zhang et al., 2020b). These data suggest that cell-intrinsic immunity in airways plays a role in restricting virus spread and constraining systemic pathology during infection. Therefore, downmodulation of IFITM proteins, and possibly other cellular proteins, by select rapalogs may contribute to the immunocompromised state that these drugs are well known to elicit in humans. This possibility warrants the investigator of the effects of different rapalog regimens on respiratory virus acquisition and disease in humans.

## Materials and Methods

### Cell lines, cell culture, inhibitors, and cytokines

HEK293T cells were obtained from ATCC (CRL-3216). HeLa-ACE2, HeLa-DPP4, and A549-ACE2 cell lines were produced by transducing cells with lentivirus packaging pWPI encoding ACE2 or DPP4 and selecting with blasticidin. HeLa IFITM1/2/3 Knockout (C5–9) cells were purchased from ATCC (CRL-3452). Primary human small airway (lung) epithelial cells (HSAEC) were purchased from ATCC (PCS-301–010). The partially immortalized nasal epithelial cell line (UNCNN2TS) was kindly provided by Scott H. Randell (University of North Carolina School of Medicine). Vero E6 cells (NR-53726) were obtained from BEI Resources. Vero-TMPRSS2 cells were a kind gift from Shan-Lu Liu (The Ohio State University). All cells were cultured at 37°C with 5% CO_2_ in Dulbecco’s Modified Eagle Medium (DMEM) supplemented with 10% fetal bovine serum (HyClone, Cytiva), except for UNCNN2TS, which were cultured in EpiX Medium (Propagenix), and HSAEC, which were cultured with airway epithelial cell basal medium (ATCC, PCS-300–030) and the bronchial epithelial cell growth kit (ATCC, PCS-300–040). Rapamycin (553211) was obtained from Sigma. Everolimus (S1120), temsirolimus (S1044), ridaforolimus (S5003), tacrolimus (S5003), and SAR405 (S7682) were obtained from Selleckchem. Type-I interferon (human recombinant interferon-beta_ser17_, NR-3085) was obtained from BEI Resources.

### Plasmids and RNA interference

pcDNA3.1 encoding human ACE2 was kindly provided by Thomas Gallagher (Loyola University). pcDNA3.1 encoding CoV-1 Spike or CoV-2 Spike tagged with a C9 epitope on the C-terminus, or MERS Spike, was kindly provided by Thomas Gallagher (Loyola University). pcDNA3.1 encoding CoV-1 Spike or CoV-2 Spike tagged with a FLAG epitope on the C-terminus was obtained from Michael Letko and Vincent Munster (NIAID). pMD2.G encoding VSV-G (12259) was obtained from Addgene (a generous gift from Didier Trono). pWPI was obtained from Addgene (12254) and human ACE2 or human TMPRSS2 was introduced by Gateway cloning (Gateway LR Clonase II Enzyme mix (11791020)) as per manufacturer’s instructions. pPolII encoding hemagglutinin (HA) or neuraminidase (NA) from Influenza A/Turkey/1/2005 (H5N1) were kindly provided by Richard Yi Tsun Kao (The University of Hong Kong). pCMV encoding HIV-1 Vpr fused to beta lactamase (pCMV4-BlaM-Vpr) was obtained from Addgene (21950). A plasmid encoding replication-incompetent HIV-1 lacking *env* and *vpr* and encoding luciferase (pNL4–3LucR-E-) was kindly provided by Vineet KewalRamani (National Cancer Institute). A plasmid encoding replication-incompetent HIV-1 lacking *env* (pNL4–3E-) was kindly provided by Olivier Schwartz (Institut Pasteur). pEGFP-N1-TFEB (38119) and pEGF-N1-Δ30TFEB (44445) were obtained from Addgene (a generous gift of Shawn M. Ferguson). pEGFP-2xFYVE (140047) was obtained from Addgene (a gift from Harald Stenmark). Silencer Select siRNA targeting IFITM3 (s195035) and a non-targeting control (No. 1) was obtained from Ambion. Cells were transfected with 20 nM siRNA using Opti-MEM (Gibco) and Lipofectamine RNAiMAX (Thermo Fisher).

### Virus and pseudovirus infections

SARS-CoV-2 isolate USA-WA1/2020 (MN985325.1) was provided by the Centers for Disease Control or by BEI Resources (NR-52281). Virus propagation was performed in Vero E6 cells. Mouse-adapted (MA) SARS-CoV-2 variant MA10 (in the USA-WA1/2020 backbone) (Leist et al., 2020) was obtained from BEI Resources (NR-55329). Virus propagation was performed in Vero E6 cells and subsequently in Vero-TMPRSS2 cells. Virus was sequenced to ensure lack of tissue culture adaptations, including furin cleavage site mutations. Virus titers were calculated by plaque assay performed in Vero E6 cells as follows: serial 10-fold dilutions were added to Vero E6 monolayers in 48-well plates for 1 hour at 37°C. Cells were overlayed with 1.5% carboxymethyl cellulose (Sigma) in modified Eagle’s medium containing 3% fetal bovine serum (Gibco), 1 mM L-glutamine, 50 units per mL penicillin and 50 μg per mL streptomycin. Three days post-infection, cells were fixed in 10% formalin and stained with crystal violet to visualize and count plaques as previously described (Chiramel et al., 2019). Titers were calculated as plaque forming units per mL and normalized as described in the figure captions. HIV-based pseudovirus was produced by transfecting HEK293T cells with 12 μg of pNL4–3LucR-E- and 4 μg of plasmid encoding viral glycoproteins (pcDNA3.1 Spike (CoV-1, CoV-2, or MERS), pMD2.G-VSV-G, or 2 μg of pPol1II-HA and 2 μg of pPol1II-NA) using TransIT-293 (Mirus). Virus supernatant was harvested 72 hours post-transfection and filtered through 0.22 μm filters. Pseudovirus titers were determined by p24 ELISA (XpressBio) and 100 ng p24 equivalent was added to target cells and incubated for 72 hours prior to lysis with Passive Lysis Buffer (Promega). Luciferase activity was measured using the Luciferase Assay System (Promega). VSV-based pseudovirus was produced as previously described (Letko et al., 2020). In brief, HEK293T cells were transfected with 2 μg pcDNA3.1 CoV-1, CoV-2, or MERS Spike using Lipofectamine2000 (Thermo Fisher). At 24 hours post-transfection, culture medium was removed from cells and 2 mL of VSV-luc/GFP + VSV-G (seed particles) was added. At 48 hours post-infection, virus supernatants were collected, clarified by centrifugation at 500×G for 5 mins, and stored. 50 μL of virus supernatants were added to target cells for a period of 24 hours prior to fixation with 4% paraformaldehyde (for measurements of GFP+ cells with flow cytometry). For infections with replication-competent SARS-CoV-2, rapamycin, everolimus, temsirolimus, or ridaforolimus (20 μM) were used to pretreat cells for 4 hours and then drugs were washed away prior to addition of virus at a multiplicity of infection (MOI) of 0.1. DMSO (Sigma) was used as a vehicle control. At one hour post-virus addition, cells were washed once with 1X PBS and overlayed with complete medium. Supernatants were harvested 24 hours later, and titers were determined on plaque assays performed in Vero E6 cells. For single-round infections using HIV- or VSV-based pseudovirus, rapamycin, everolimus, temsirolimus, ridaforolimus, or tacrolimus (20 μM) were used to pretreat cells for 4 hours and were maintained for the duration of infection and until harvest of cells for luciferase assay or flow cytometry. DMSO (Sigma) was used as a vehicle control.

### FRET-based virus entry assay

HIV-based pseudovirus incorporating BlaM-Vpr and CoV-2 Spike was produced by transfecting HEK293T cells with pNL4–3E- (15 μg), pCMV4-BlaM-Vpr (5 μg), and pcDNA3.1 CoV-2 Spike (5 μg) using the calcium phosphate technique. Briefly, six million 293T cells were seeded in a T75 flask. Plasmid DNA was mixed with sterile H2O, CaCl_2_, and Tris-EDTA (TE) buffer, and the totality was combined with Hepes-buffered saline (HBS). The transfection volume was added dropwise, and cells were incubated at 37°C for 48 h. Supernatants were recovered and clarified by centrifugation, passed through a 0.45 μm filter, and stored. Titers were measured using an HIV-1 p24 ELISA kit (XpressBio). 50 ng p25 equivalent of virus was added to HeLa-ACE2 cells for 2 hours. Cells were washed and labeled with the CCF2-AM β-lactamase Loading Kit (Invitrogen) for 2 hours and analyzed for CCF2 cleavage by flow cytometry as described (Compton et al., 2014). Rapamycin, everolimus, temsirolimus, or ridaforolimus (20 μM) were used to pretreat cells for 4 hours prior to virus addition and were maintained for the duration of infection. DMSO (Sigma) was used as a vehicle control.

### Western blot, flow cytometry, and antibodies

Whole cell lysis was performed with RIPA buffer (Thermo Fisher) supplemented with Halt Protease Inhibitor EDTA-free (Thermo Fisher). Lysates were clarified by centrifugation and supernatants were collected and stored. Protein concentration was determined with the Protein Assay Kit II (Bio-Rad), and 10–15 μg of protein was loaded into 12% acrylamide Criterion XT Bis-Tris Precast Gels (Bio-Rad). Electrophoresis was performed with NuPage MES SDS Running Buffer (Invitrogen) and proteins were transferred to Amersham Protran Premium Nitrocellulose Membrane, pore size 0.20 μm (GE Healthcare). Membranes were blocked with Odyssey Blocking Buffer (Li-COR) and incubated with the following primary antibodies diluted in Odyssey Antibody Diluent (Li-COR): anti-IFITM1 (60074–1-Ig; Proteintech), anti-IFITM2 (66137–1-Ig; Proteintech), anti-IFITM3 (EPR5242, ab109429; Abcam), anti-IFITM2/3 (66081–1-Ig; Proteintech), anti-actin (C4, sc-47778; Santa Cruz Biotechnology), anti-hACE2 (ab15348; Abcam), anti-TFEB (4240S; Cell Signaling Technology), and anti-pTFEB (Ser211) (37681S; Cell Signaling Technology). Secondary antibodies conjugated to DyLight 800 or 680 (Li-Cor) and the Li-Cor Odyssey CLx imaging system were used to reveal specific protein detection. Images were analyzed (including signal quantification) and assembled using ImageStudioLite (Li-Cor).

### Confocal fluorescence and immunofluorescence microscopy

HeLa-ACE2 cells were fixed with 4% paraformaldehyde, stained with anti-IFITM2/3 (66081–1-Ig; Proteintech), goat anti-mouse IgG Alexa Fluor 647 (A21235; Thermo Fisher) and DAPI (62248; Thermo Fisher), and imaged in a glass-bottom tissue culture plate with an Operetta CLS High-Content Analysis System (Perkin Elmer). For measurement of TFEB-GFP nuclear/cytoplasmic distribution, HeLa-ACE2 cells were transfected with pEGFP-N1-TFEB for 24 hours, fixed with 4% paraformaldehyde, stained with HCS CellMask Red Stain (H32712; Thermo Fisher) and DAPI, and imaged with an Operetta CLS. Using Harmony software (Perkin Elmer), nuclear/cytoplasmic ratios of TFEB-GFP were calculated in single cells as follows: cells were delineated by CellMask Red Stain, nuclei were delineated by DAPI, nuclear TFEB-GFP was designated as GFP overlapping with DAPI, and cytoplasmic TFEB-GFP was designated as total GFP signal minus nuclear TFEB-GFP. Average ratios were calculated from 20–30 cells per field, and the mean of averages from 10 fields was obtained (total of approximately 250 cells per condition). For measurement of IFITM2/3 levels in cells transfected with TFEBΔ30-GFP, HeLa-ACE2 cells were transfected with pEGF-N1-Δ30TFEB for 24 hours, fixed and permeabilized with BD Cytofix/Cytoperm (Fisher Scientific), stained with anti-IFITM2/3 and goat anti-mouse IgG Alexa Fluor 647, and imaged with an Operetta CLS. The IFITM2/3 fluorescence intensity within a single, medial Z section was measured in approximately 150 GFP-negative cells and 150 GFP-positive cells using the freehand selections tool in ImageJ.

### Proteomics by mass spectrometry

Protein Digestion and TMT labeling. Cell pellets were produced in triplicate from A549-ACE2 cells treated with 20 μM temsirolimus or ridaforolimus, or an equivalent volume of DMSO and lysed in RIPA buffer followed by sonication. Lysates were clarified by centrifugation and protein concentration was quantified using BCA protein estimation kit (Thermo Fisher). One hundred micrograms of lysate were alkylated and digested by addition of trypsin at a ratio of 1:50 (Promega) and incubating overnight at 37°C. Digestion was acidified by adding formic acid (FA) to a final concentration of 1% and desalted using peptide desalting columns (Thermo Fisher) according to manufacturer’s protocol. Peptides were eluted from the columns using 50% ACN/0.1% FA, dried in a speedvac, and kept frozen at −20°C until further analysis. For TMT labeling, 15 μg of each sample was reconstituted in 50 μL of 50 mM HEPES, pH 8.0, and 75 μg of TMTpro label (Thermo Fisher) in 100% ACN was added to each sample. After incubating the mixture for 1 hr at room temperature with occasional mixing, the reaction was terminated by adding 8 μL of 5% hydroxylamine. The peptide samples for each condition were pooled and cleaned using peptide desalting columns (Thermo Fisher). High pH reverse phase fractionation. The first dimensional separation of the peptides was performed using a Waters Acquity UPLC system coupled with a fluorescence detector (Waters) using a 150 mm × 3.0 mm Xbridge Peptide BEM^™^ 2.5 um C18 column (Waters) operating at 0.35 mL/min. The dried peptides were reconstituted in 100 μL of mobile phase A solvent (3 mM ammonium bicarbonate, pH 8.0). Mobile phase B was 100% acetonitrile (Thermo Fisher). The column was washed with mobile phase A for 10 min followed by gradient elution 0–50% B (10–60 min) and 50–75% B (60–70 min). The fractions were collected every minute. These 60 fractions were pooled into 24 fractions. The fractions were vacuum centrifuged to dryness and stored at −80°C until analysis by mass spectrometry. Mass Spectrometry acquisition and data analysis. The dried peptide fractions were reconstituted in 0.1% TFA and subjected to nanoflow liquid chromatography (Thermo Ultimate^™^ 3000RSLC nano LC system, Thermo Scientific) coupled to an Orbitrap Eclipse mass spectrometer (Thermo Scientific). Peptides were separated using a low pH gradient using 5–50% ACN over 120 minutes in mobile phase containing 0.1% formic acid at 300 nL/min flow rate. MS scans were performed in the Orbitrap analyzer at a resolution of 120,000 with an ion accumulation target set at 4e^5^ and max IT set at 50ms over a mass range of 400–1600 m/z. Ions with determined charge states between 2 and 5 were selected for MS2 scans in the ion trap with CID fragmentation (Turbo; NCE 35%; maximum injection time 35 ms; AGC 1 × 10^4^). The spectra were searched using the Real Time Search Node in the tune file using human Uniprot database using Comet search algorithm with TMT16 plex (304.2071Da) set as a static modification of lysine and the N-termini of the peptide. Carbamidomethylation of cysteine residues (+57.0214 Da) was set as a static modification, while oxidation of methionine residues (+15.9949 Da) was set up as dynamic modification. For the selected peptide, an SPS–MS3 scan was performed using up to 10 *b*- and *y*-type fragment ions as precursors in an Orbitrap at 50,000 resolution with a normalized AGC set at 500 followed by maximum injection time set as “Auto” with a normalized collision energy setting of 65. Acquired MS/MS spectra were searched against a human Uniprot protein database along with a contaminant protein database, using a SEQUEST and percolator validator algorithms in the Proteome Discoverer 2.4 software (Thermo Scientific). The precursor ion tolerance was set at 10 ppm and the fragment ions tolerance was set at 0.02 Da along with methionine oxidation included as dynamic modification. Carbamidomethylation of cysteine residues and TMT16 plex (304.2071Da) was set as a static modification of lysine and the N-termini of the peptide. Trypsin was specified as the proteolytic enzyme, with up to 2 missed cleavage sites allowed. Searches used a reverse sequence decoy strategy to control for the false peptide discovery and identifications were validated using percolator software. Reporter ion intensities were adjusted to correct for the impurities according to the manufacturer’s specification and the abundances of the proteins were quantified using the summation of the reporter ions for all identified peptides. The reporter abundances were normalized across all the channels to account for equal peptide loading. Median abundance values for DMSO, temsirolimus, and ridaforolimus were calculated from three replicates and presented as medians plus 95% confidence intervals.

### In vivo infections of hamsters and mice with SARS-CoV-2

Male Golden Syrian hamsters between the ages of 6–8 weeks were acclimated for 11 days following receipt. Hamsters received an intraperitoneal injection (500 μL) of rapamycin (HY-10219; MedChemExpress) or ridaforolimus (HY-50908; MedChemExpress) at 3 mg/kg or an equivalent amount of DMSO (4 hamsters per group). Four hours later, hamsters were challenged with 6 × 10^3^ plaque forming units of SARS-CoV-2 isolate USA-WA1/2020 (amplified on Calu-3 cells) through intranasal inoculation (50 μL in each nare). Clinical observations and weights were recorded daily up until day 10 post-infection. According to Institutional Animal Care and Use Committee human euthanasia criteria, hamsters were euthanized immediately if weight loss exceeded 20%. Otherwise, hamsters were euthanized on day 10 post-infection. Oral swabs were collected for measurement of viral RNA by quantitative PCR of the viral N (nucleocapsid) gene. Lungs were harvested following euthanasia (day 10 or earlier) and infectious viral load was determined by TCID_50_ assay in Vero-TMPRSS2 cells. C57BL/6 mice received an intraperitoneal injection of rapamycin (NC9362949; LC-Laboratories) or an equivalent amount of DMSO (7 and 6 mice per group, respectively). The following day, mice were challenged intranasally with 6 × 10^4^ TCID_50_ equivalent of MA10 SARS-CoV-2 (USA-WA1/2020 backbone). Mice received a second injection of rapamycin or DMSO on the day of infection and a third on day one post-infection. Mice were euthanized for lung harvest on day two post-infection. Infectious viral load was determined by TCID_50_ assay in Vero-TMPRSS2 cells. Animal studies were conducted in compliance with all relevant local, state, and federal regulations and were approved by the Institutional Animal Care and Use Committee of Bioqual and of the Ohio State University.

## Supplementary Material

Supplement 1 **Supplemental File 1:** Spreadsheet of proteomics data obtained by mass spectrometry.

1 **Supplemental Figure 1:** (A) Primary HSAEC were treated with 20 μM Rap, Eve, Tem, Rid, or an equivalent volume of DMSO for 4 hours and whole cell lysates were subjected to SDS-PAGE and Western blot analysis. Immunoblotting was performed with anti-IFITM2 (not detected), anti-IFITM3, anti-IFITM1, and anti-actin. (B) Primary HSAEC were transfected with siRNA targeting IFITM3 or control siRNA for 48 hours. VSV-CoV-2 (50 μL) was added to cells and infection was measured by GFP expression at 24 hours post-infection using flow cytometry. siRNA-transfected cells were subjected to SDS-PAGE and Western blot analysis. Immunoblotting was performed with anti-IFITM2 (not detected), anti-IFITM3, anti-IFITM1, and anti-actin. (C) Semi-transformed nasal epithelial cells (UNCNNTS2) were treated with 20 μM Rap, Eve, Tem, Rid, or an equivalent volume of DMSO for 4 hours and whole cell lysates were subjected to SDS-PAGE and Western blot analysis. Immunoblotting was performed with anti-IFITM2 (not detected), anti-IFITM3, anti-IFITM1, and anti-actin. Immunoblots are representative of 3 independent experiments. Means and standard error were calculated from 3 experiments. Statistical analysis was performed with student’s T test and asterisks indicate significant difference from control siRNA. *, p < 0.05; **, p < 0.01. Rel.; relative.
**Supplemental Figure 2:** (A) HeLa-ACE2 were treated with 20 μM Rap, Eve, Tem, Rid, or an equivalent volume of DMSO for 24 hours and whole cell lysates were subjected to SDS-PAGE and Western blot analysis. Immunoblotting was performed with anti-IFITM2/3 and anti-actin. (B) HeLa-ACE2 were treated with 20 μM Rap, Eve, Tem, Rid, or an equivalent volume of DMSO in the presence or absence of 1 μM Bafilomycin A1 for 4 hours and whole cell lysates were subjected to SDS-PAGE and Western blot analysis. Immunoblotting was performed with anti-IFITM2, anti-IFITM1, anti-IFITM3, and anti-actin (in that order) on the same nitrocellulose membrane. (C) HeLa-ACE2 cells were transected with FYVE-GFP for 24 hours followed by treatment with 100 nM SAR405 or an equivalent volume of ethanol (vehicle) for 3 hours. Cells were fixed and imaged by confocal immunofluorescence microscopy. For each condition, a Z-stack of 25 slices is shown as a maximum intensity projection. (D) HeLa-ACE2 were treated with 20 μM Rap, Eve, Tem, Rid, or an equivalent volume of DMSO in the presence or absence of 100 nM SAR405 for 4 hours and whole cell lysates were subjected to SDS-PAGE and Western blot analysis. Immunoblotting was performed with anti-IFITM2/3 and anti-actin on the same nitrocellulose membrane. All immunoblots are representative of three independent experiments.
**Supplemental Figure 3:** (A) A549-ACE2 were treated with 20 μM Rap, Eve, Tem, Rid, or Tac for 4 hours and whole cell lysates were subjected to SDS-PAGE and Western blot analysis. Immunoblotting was performed with anti-TFEB and anti-pTFEB (S211). (B) pTFEB (S211) levels were divided by total TFEB levels and summarized as an average of 3 experiments. Means and standard error are shown. Statistical analysis was performed with one-way ANOVA and asterisks indicate significant difference from control siRNA. (C) A549-ACE2 cells were treated with 20 μM Tem, Rid, or an equivalent volume of DMSO for 4 hours and proteins were extracted for analysis by mass spectrometry. Raw data in triplicate of all proteins detected is presented in Supplemental File 1. Median protein levels for five genes known to be regulated by TFEB are shown. ACE2 is also shown for comparison. Statistical analysis was performed with a student’s T test and asterisks indicate significant difference from DMSO. *, p < 0.05 or p = 0.05; **, p < 0.01. Rel.; relative.
