# Supplementary figures and images for: Rapalogs downmodulate intrinsic immunity and promote cell entry of SARS-CoV-2
Source: bioRxiv. 2022 Apr 6:2021.04.15.440067. Originally published 2021 Apr 16. Preprint. [Version 4] doi: 10.1101/2021.04.15.440067 (PMC8057238; doi:10.1101/2021.04.15.440067)

Supplemental Figure 1

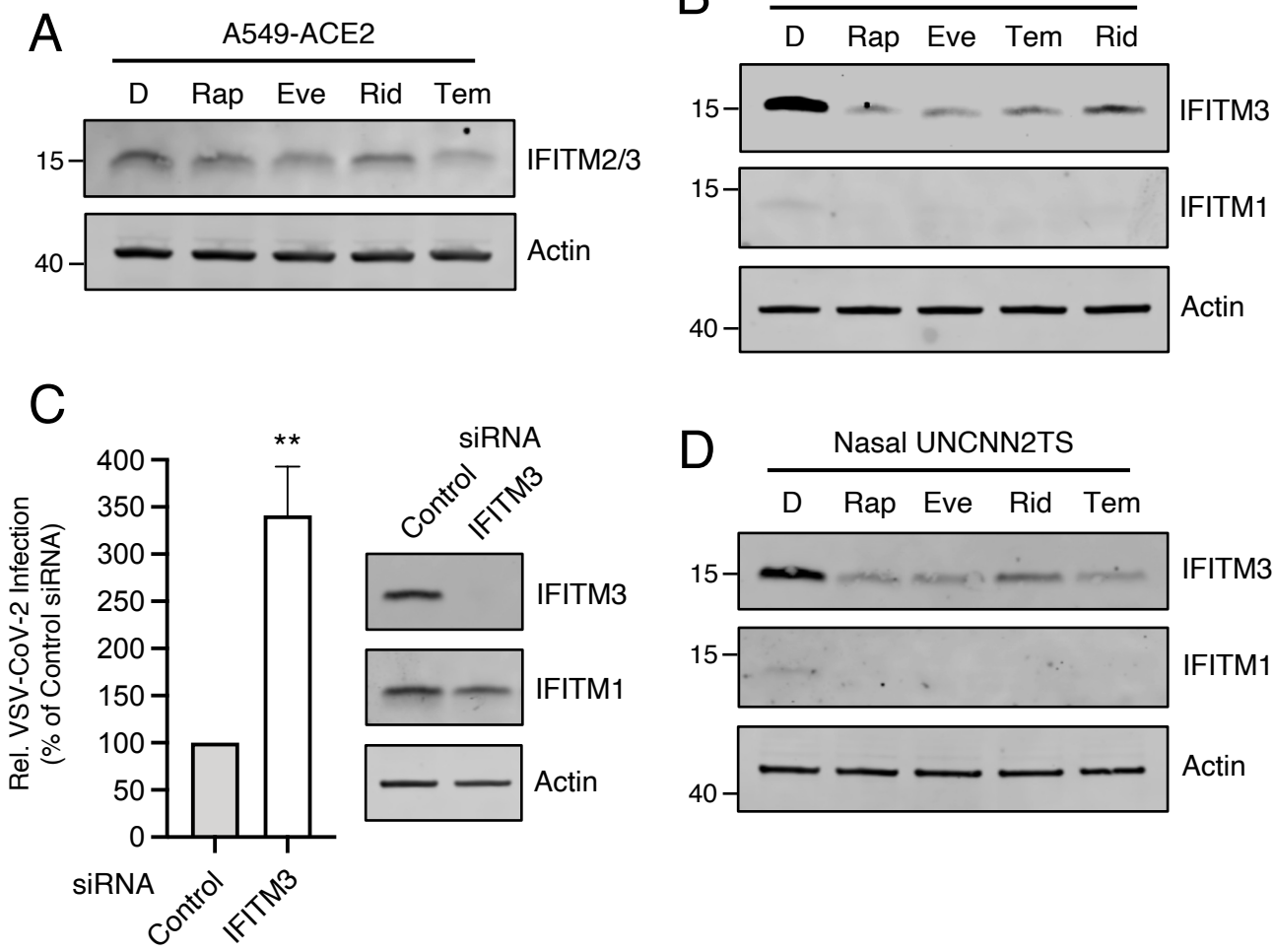

Supplement: Supplement 1 — Supplemental Figure 1: (A) A549-ACE2 cells were treated with 20 μM Rap, Eve, Rid, Tem, or an equivalent volume of DMSO (in the absence of type-I interferon) for 4 hours and whole cell lysates were subjected to SDS-PAGE and Western blot analysis. Immunoblotting was performed with anti-IFITM2/3 and anti-actin. (B) Primary HSAEC were treated with 20 μM Rap, Eve, Tem, Rid, or an equivalent volume of DMSO for 4 hours and whole cell lysates were subjected to SDS-PAGE and Western blot analysis. Immunoblotting was performed with anti-IFITM2 (not detected), anti-IFITM3, anti-IFITM1, and anti-actin. (C) Primary HSAEC were transfected with siRNA targeting IFITM3 or control siRNA for 48 hours. VSV-CoV-2 (50 μL) was added to cells and infection was measured by GFP expression at 24 hours post-infection using flow cytometry. siRNA-transfected cells were subjected to SDS-PAGE and Western blot analysis. Immunoblotting was performed with anti-IFITM2 (not detected), anti-IFITM3, anti-IFITM1, and anti-actin. (D) Semi-transformed nasal epithelial cells (UNCNN2TS) were treated with 20 μM Rap, Eve, Tem, Rid, or an equivalent volume of DMSO for 4 hours and whole cell lysates were subjected to SDS-PAGE and Western blot analysis. Immunoblotting was performed with anti-IFITM2 (not detected), anti-IFITM3, anti-IFITM1, and anti-actin. Immunoblots are representative of 3 independent experiments. Means and standard error were calculated from 3 experiments. Statistical analysis was performed with student’s T test and asterisks indicate significant difference from control siRNA. *, p < 0.05; **, p < 0.01. Rel.; relative. [file media-1.pdf]

**A**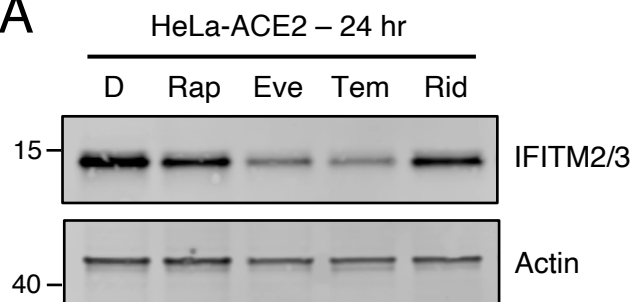**B**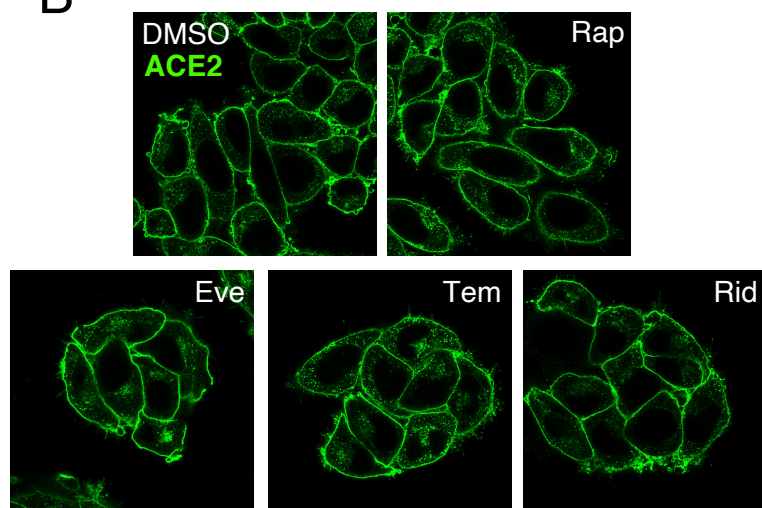**C**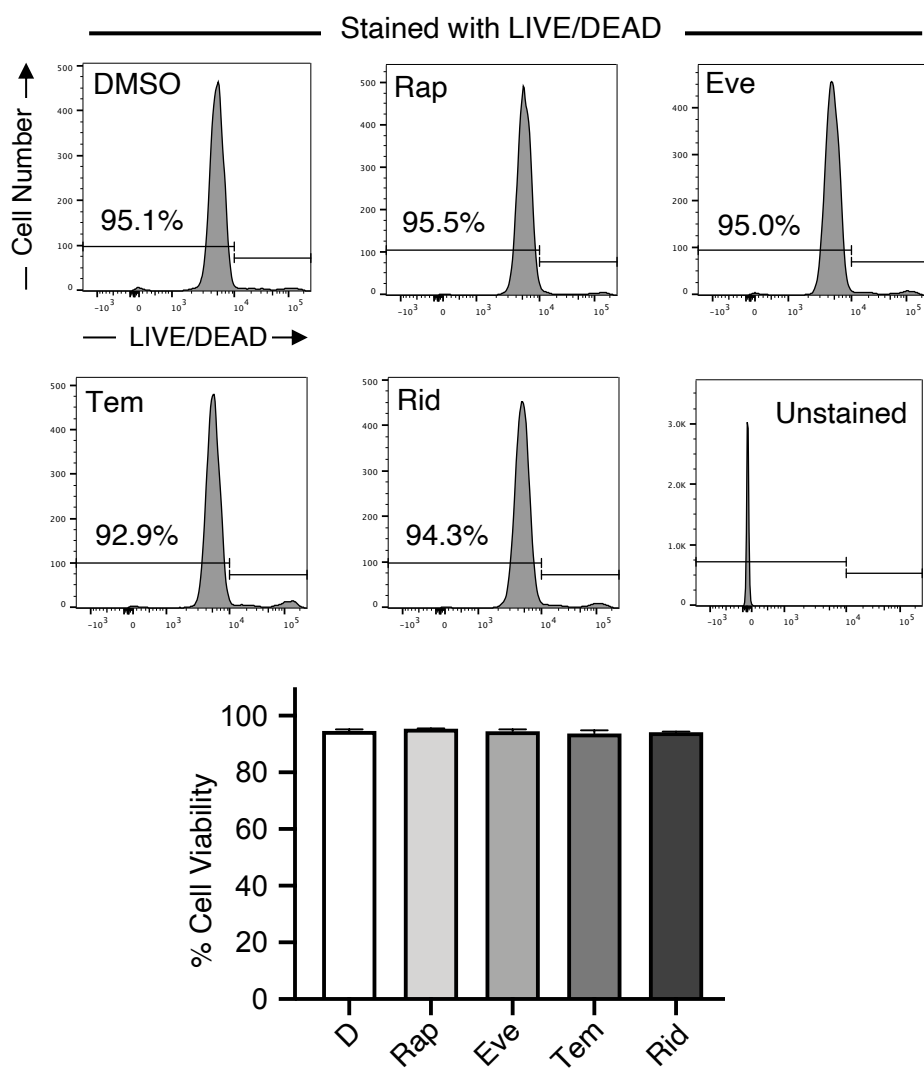

Supplement: Supplement 2 — Supplemental Figure 2: (A) HeLa-ACE2 were treated with 20 μM Rap, Eve, Tem, Rid, or an equivalent volume of DMSO for 24 hours and whole cell lysates were subjected to SDS-PAGE and Western blot analysis. Immunoblotting was performed with anti-IFITM2/3 and anti-actin. (B) HeLa-ACE2 were treated with 20 μM Rap, Eve, Tem, Rid, or an equivalent volume of DMSO, in the presence or absence of 1 μM Bafilomycin A1, for 4 hours and whole cell lysates were subjected to SDS-PAGE and Western blot analysis. Immunoblotting was performed with anti-IFITM2, anti-IFITM1, anti-IFITM3, and anti-actin (in that order) on the same nitrocellulose membrane. (C) HeLa-ACE2 cells were transected with FYVE-GFP for 24 hours followed by treatment with 100 nM SAR405 or an equivalent volume of ethanol (vehicle) for 3 hours. Cells were fixed and imaged by confocal immunofluorescence microscopy. For each condition, a Zstack of 25 slices is shown as a maximum intensity projection. (D) HeLa-ACE2 were treated with 20 μM Rap, Eve, Tem, Rid, or an equivalent volume of DMSO in the presence or absence of 100 nM SAR405 for 4 hours and whole cell lysates were subjected to SDS-PAGE and Western blot analysis. Immunoblotting was performed with anti-IFITM2/3 and anti-actin on the same nitrocellulose membrane. (E) HeLa-ACE2 were treated with 20 μM Rap, Eve, Tem, Rid, or an equivalent volume of DMSO in the presence of 1 μM Bafilomycin A1, 5 μg/mL U18666A, or neither, for 4 hours. Cells were then fixed, permeabilized, and stained with anti-IFITM2/3. IFITM2/3 protein levels were measured using flow cytometry. Means and standard error were calculated from 3 experiments. Statistical analysis was performed with one-way ANOVA and asterisks indicate significant difference from DMSO. *, p < 0.05; **, p < 0.01. Rel.; relative. All immunoblots are representative of three independent experiments. [file media-2.pdf]

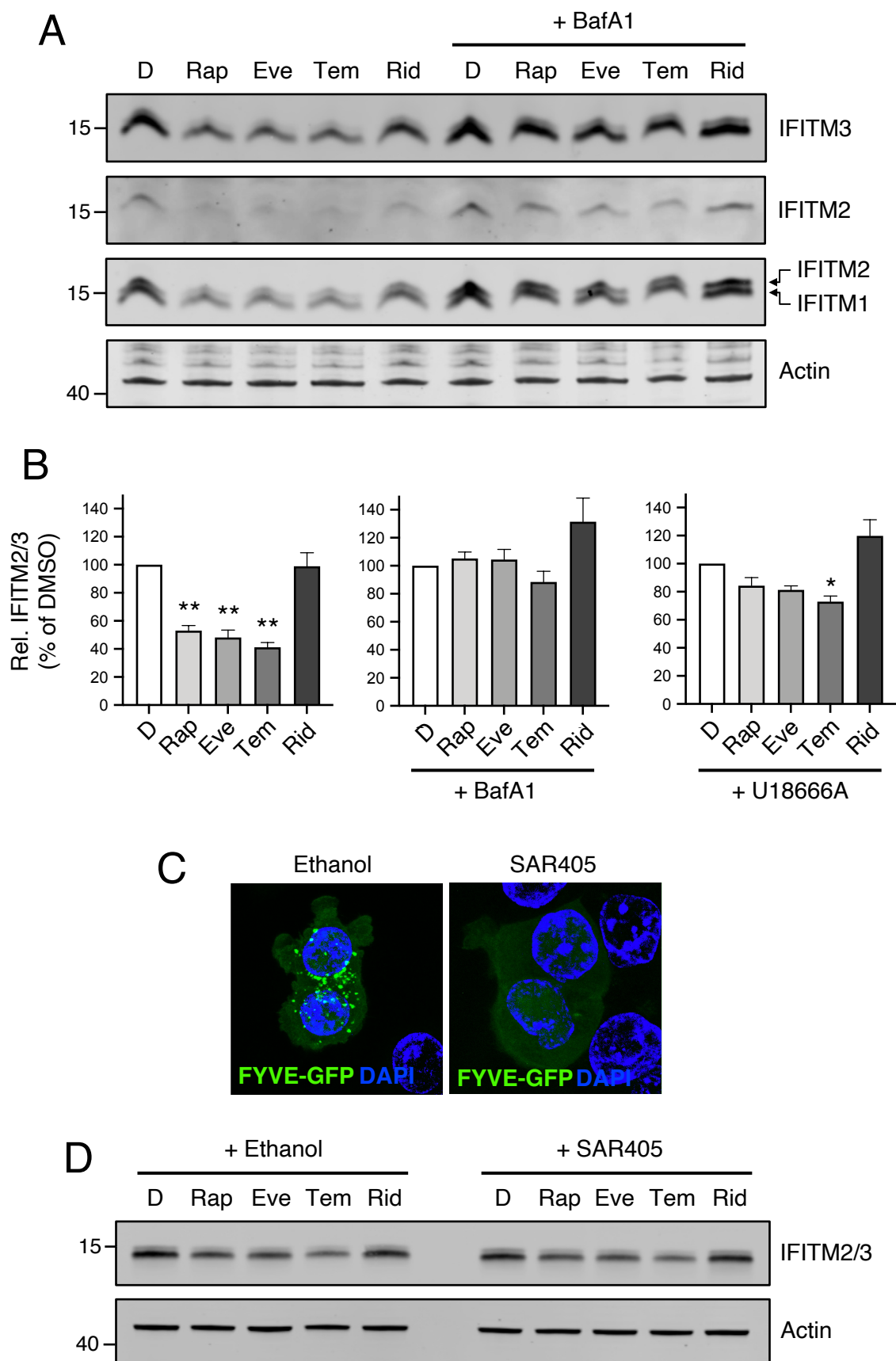

Supplement: Supplement 3 — Supplemental Figure 3: (A) HeLa-ACE2 cells were transfected with 0.3 μg pcDNA3.1-hACE2 for 24 hours and treated with 20 μM Rap, Eve, Tem, Rid, or the equivalent volume of DMSO for 4 hours and whole cell lysates were subjected to SDS-PAGE and Western blot analysis. Cells were fixed, permeabilized, stained with anti-ACE2, and imaged by confocal immunofluorescence microscopy. Images represent a single, medial Z section. (B) HeLa-ACE2 cells were treated with 20 μM Rap, Eve, Tem, Rid, or the equivalent volume of DMSO for 4 hours and subsequently fixed and stained with LIVE/DEAD Fixable Red Dead Cell Stain Kit for 30 minutes according to manufacturer’s instructions. Cells were analyzed by flow cytometry. Means and standard error were calculated from 2 experiments. Statistical analysis was performed with one-way ANOVA and asterisks indicate significant difference from DMSO. *, p < 0.05; **, p < 0.01. Rel.; relative. [file media-3.pdf]

Supplemental Figure 4

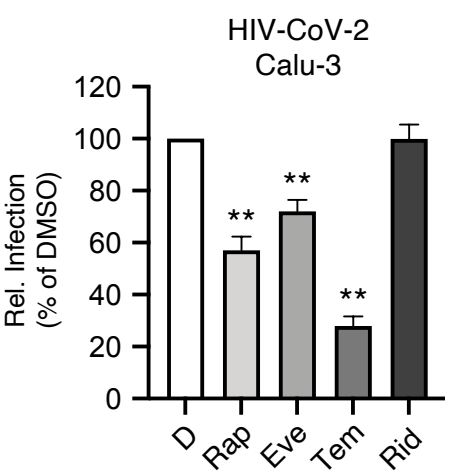

Supplement: Supplement 4 — Supplemental Figure 4: Calu-3 cells were treated with 20 μM Rap, Eve, Tem, Rid, or the equivalent volume of DMSO for 4 hours. HIV-CoV-2 (100 ng p24 equivalent) was added to cells and infection was measured by luciferase activity at 48 hours post-infection. Luciferase units were normalized to 100 in the DMSO condition. Means and standard error were calculated from 3 experiments. Statistical analysis was performed with one-way ANOVA and asterisks indicate significant difference from DMSO. *, p < 0.05; **, p < 0.01. Rel.; relative. [file media-4.pdf]

Supplemental Figure 5

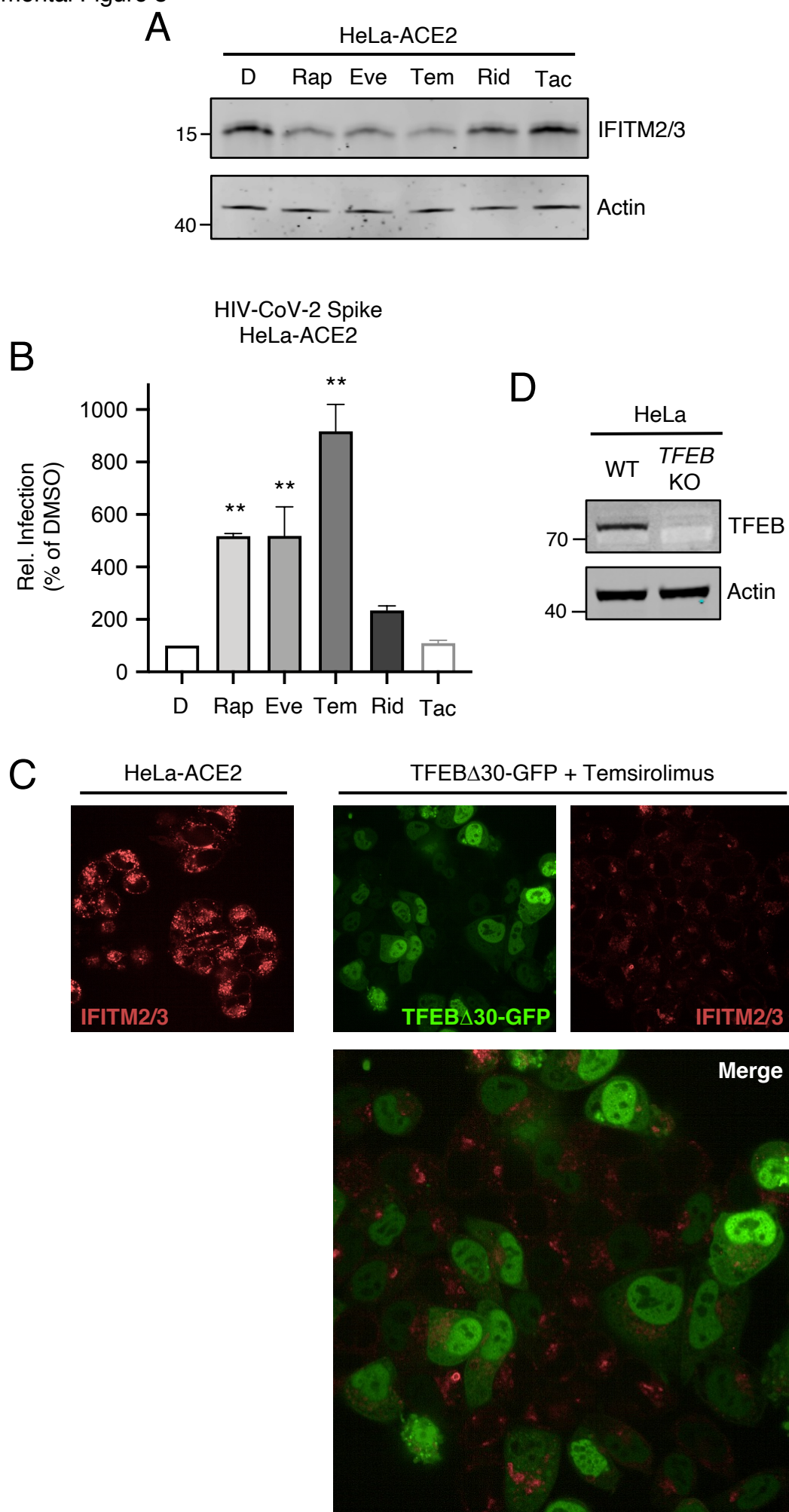

Supplement: Supplement 5 — Supplemental Figure 5: (A) HeLa-ACE2 cells were treated with 20 μM Rap, Eve, Tem, Rid, Tac, or the equivalent volume of DMSO for 4 hours. Whole cell lysates were subjected to SDS-PAGE and Western blot analysis. Immunoblotting was performed with anti-IFITM2/3 and anti-actin on the same nitrocellulose membrane. (B) HeLa-ACE2 cells were treated with 20 μM Rap, Eve, Tem, Rid, Tac, or the equivalent volume of DMSO for 4 hours. HIV-CoV-2 (100 ng p24 equivalent) was added to cells and infection was measured by luciferase activity at 48 hours post-infection. Luciferase units were normalized to 100 in the DMSO condition. Means and standard error were calculated from 3 experiments. Statistical analysis was performed with one-way ANOVA and asterisks indicate significant difference from DMSO. *, p < 0.05; **, p < 0.01. Rel.; relative. (C) HeLa-ACE2 were transfected with 0.5 μg TFEBΔ30-GFP for 24 hours and treated with 20 μM Tem for four hours. Cells were then fixed, permeabilized, stained with anti-IFITM2/3, and imaged by confocal immunofluorescence microscopy. Representative images are shown and anti-IFITM2/3 staining in untreated HeLa-ACE2 are shown for comparison. (D) Whole cell lysates from HeLa WT and HeLa TFEB KO cells were subjected to SDS-PAGE and Western blot analysis. Immunoblotting was performed with anti-TFEB and anti-actin on the same nitrocellulose membrane. [file media-5.pdf]

Supplemental Figure 6

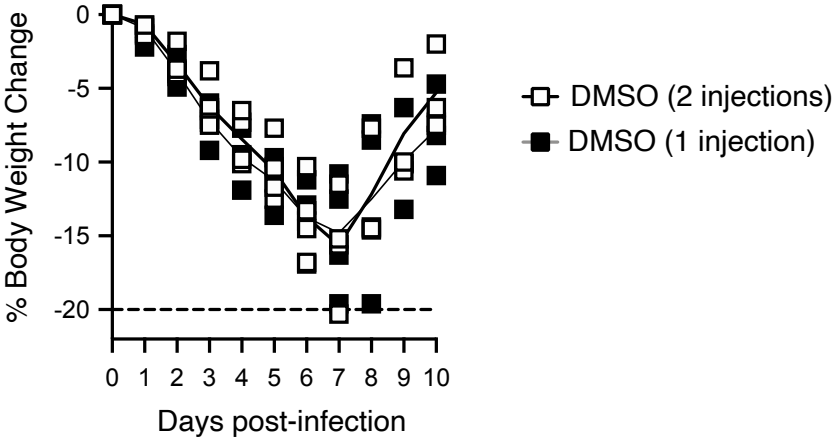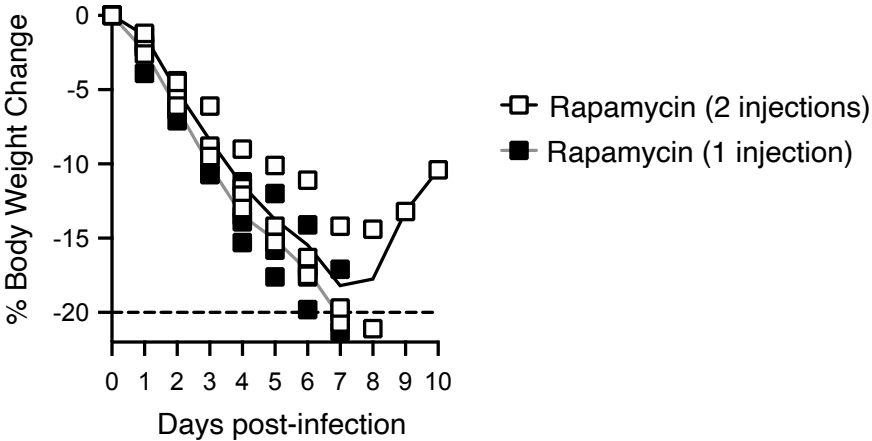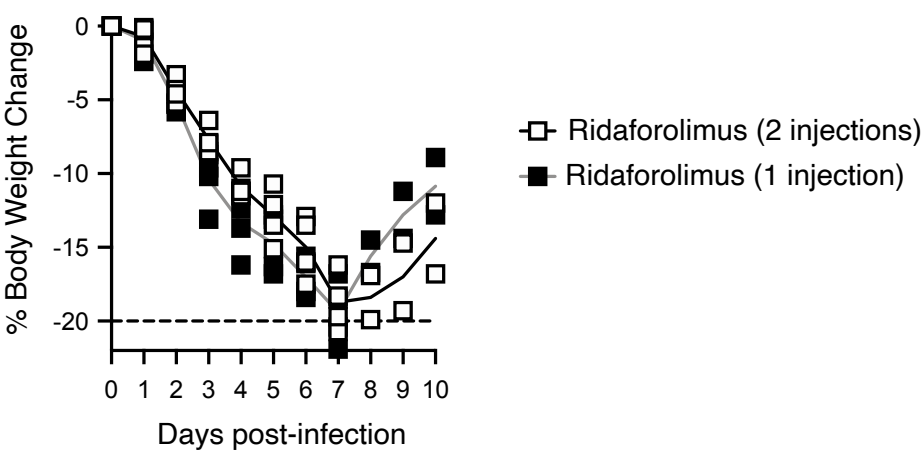

Supplement: Supplement 6 — Supplemental Figure 6: Body weight measurements for individual hamsters following injections with DMSO (A), Rap (B), or Rid (C) are plotted by day post-infection and presented as % body weight change relative to Day 0. Hamsters receiving one injection of 3 mg/kg DMSO, Rap, or Rid prior to infection (n=4, 1 injection) are indicated by black squares, while hamsters receiving one injection prior to infection as well as a second injection of 3 mg/kg DMSO, Rap, or Rid at Day 2 post-infection (n=4, 2 injections) are indicated by white squares. The average daily weight change for each group is indicated by grey and black lines, respectively. If and when a hamster lost 20% or more of its body weight, it was euthanized and body weight measurements were stopped. [file media-6.pdf]
