# Supplementary material for: Rapalogs downmodulate intrinsic immunity and promote cell entry of SARS-CoV-2
Source: bioRxiv. 2022 Jan 6:2021.04.15.440067. Originally published 2021 Apr 16. Preprint. [Version 3] doi: 10.1101/2021.04.15.440067 (PMC8057238; doi:10.1101/2021.04.15.440067)

Supplemental Figure 1

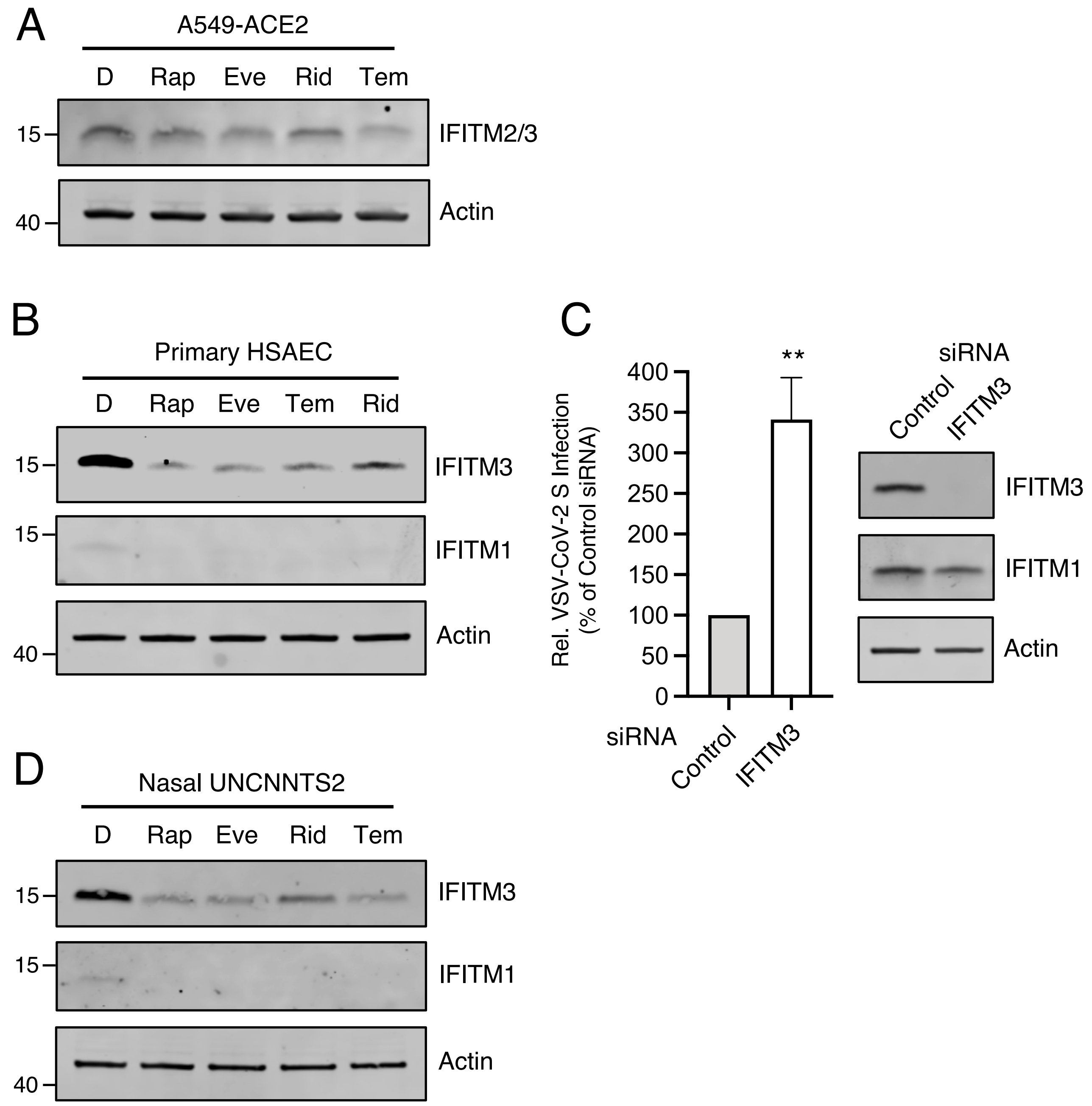

Supplemental Figure 2

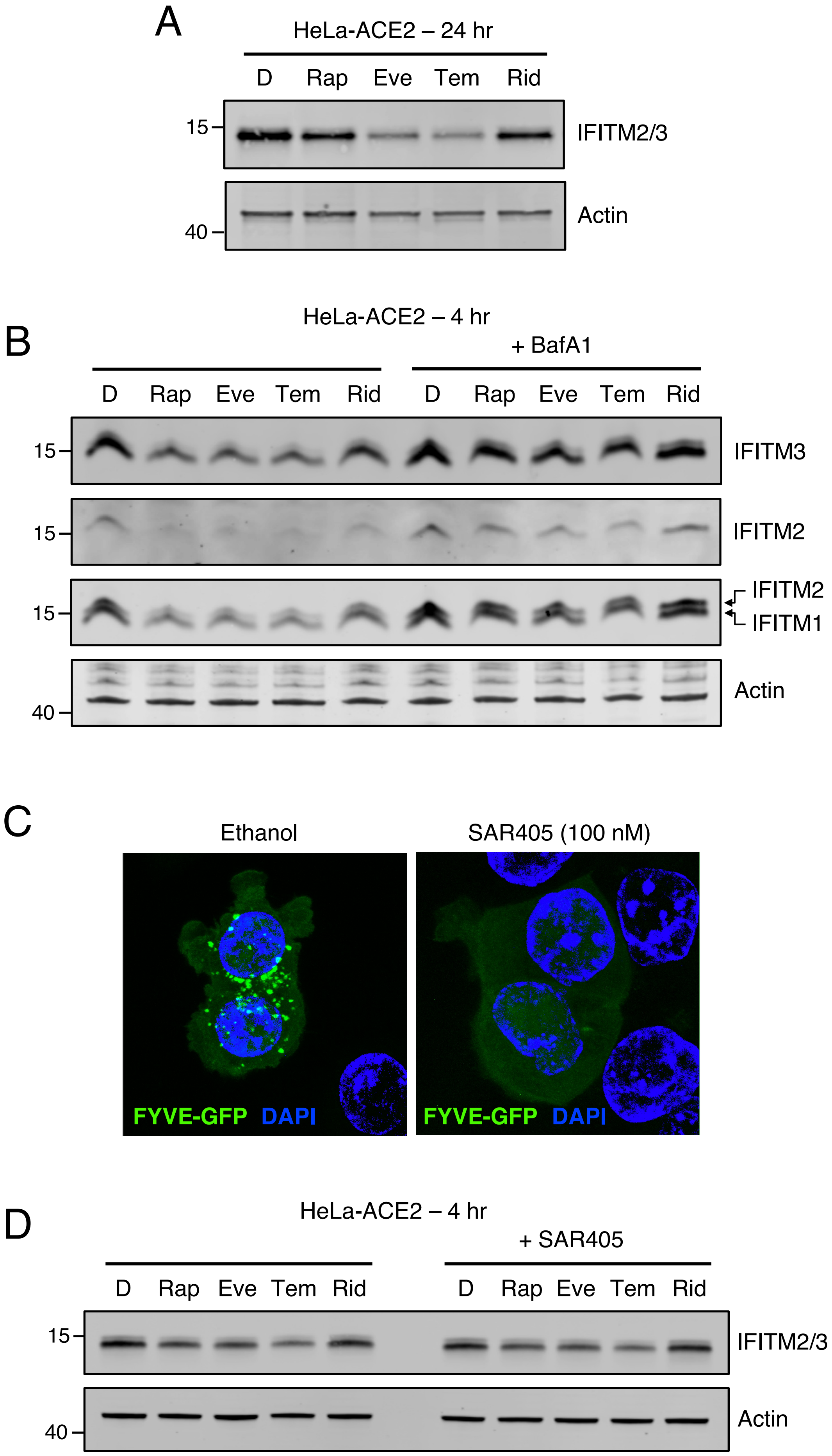

Supplemental Figure 3

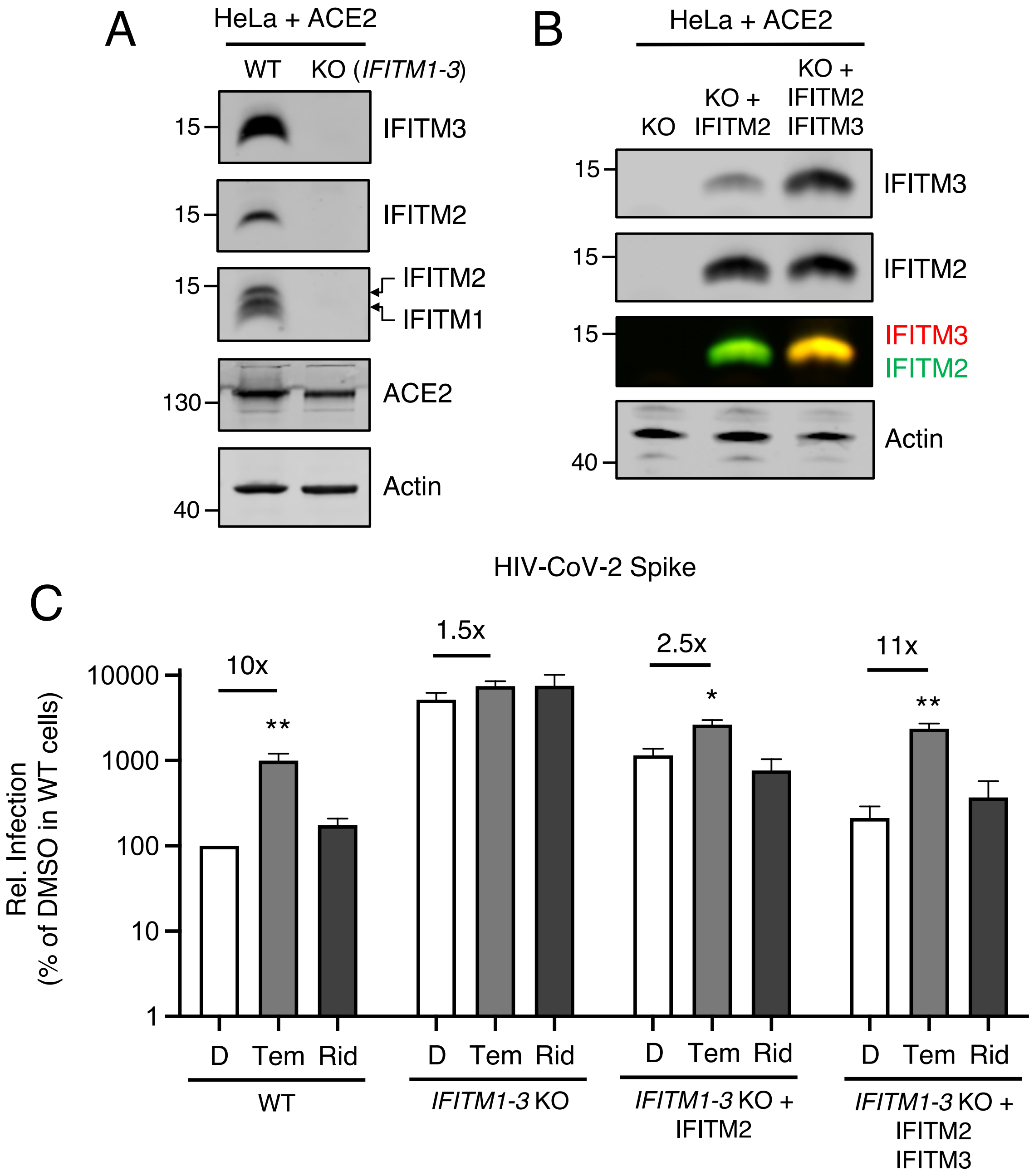

Supplemental Figure 4

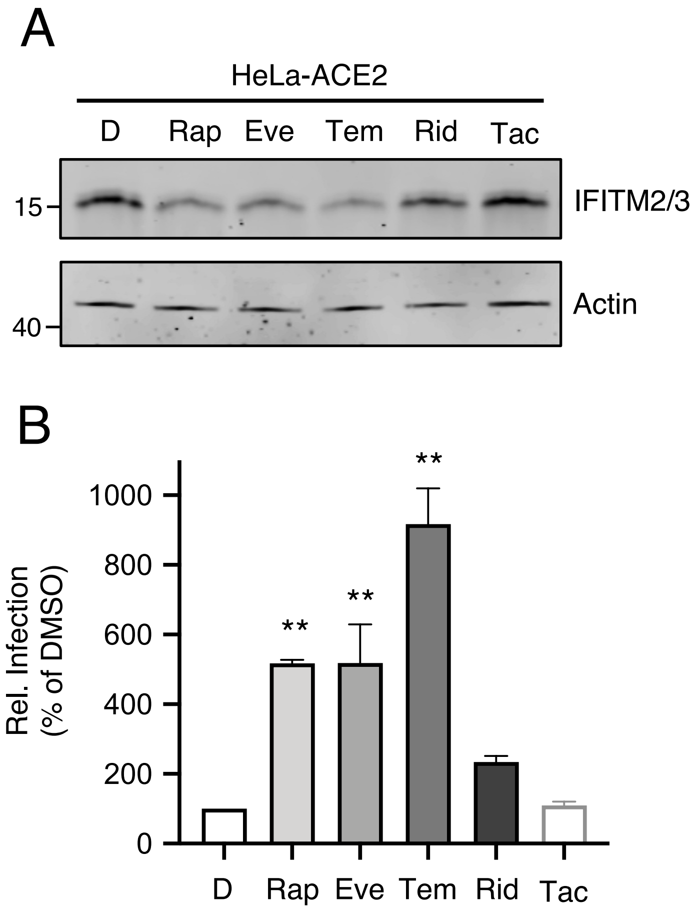

Supplement: Supplementary file 1 [file NIHPP2021.04.15.440067V3-supplement-1.pdf]
