# Supplementary material for: Rapalogs downmodulate intrinsic immunity and promote cell entry of SARS-CoV-2
Source: bioRxiv. 2021 Apr 16:2021.04.15.440067. Preprint. [Version 1] doi: 10.1101/2021.04.15.440067 (PMC8057238; doi:10.1101/2021.04.15.440067)
Supplement: Supplementary file 1 [file NIHPP2021.04.15.440067-supplement-1.pdf]

bioRxiv preprint doi: https://doi.org/10.1101/2021.04.15.440067; this version posted April 16, 2021. The copyright holder for this preprint (which was not certified by peer review) is the author/funder. This article is a US Government work. It is not subject to copyright under 17 USC 105 and is also made available for use under a CC0 license.

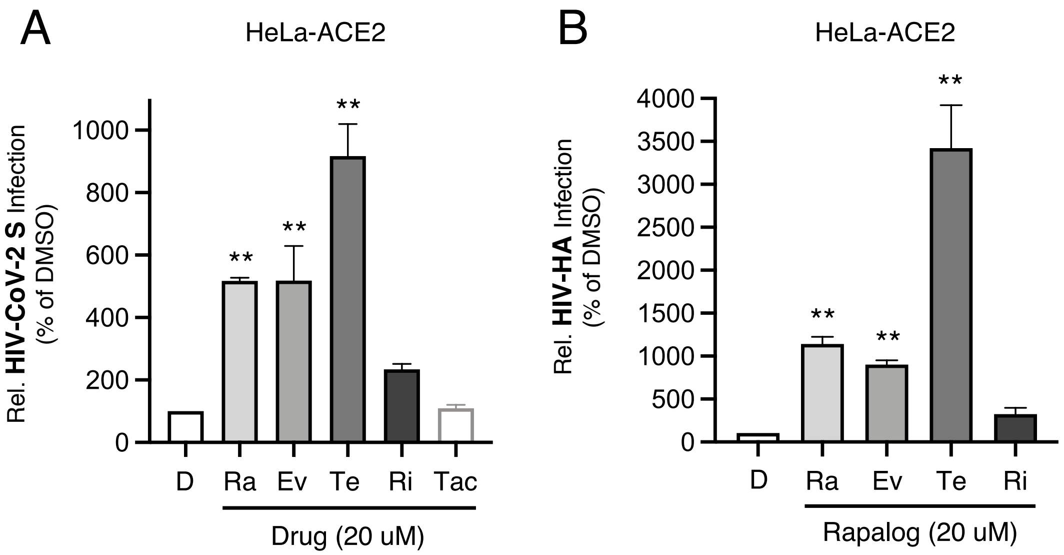

Supplemental Figure 1:
(A) HeLa-ACE2 cells were treated with 20 µM of rapamycin (Ra), everolimus (Ev), temsirolimus (Te), ridaforolimus (Ri), tacrolimus (Tac), or an equivalent volume of DMSO (D) for 4 hours and subsequently exposed to HIV-CoV-2 S pseudovirus. Cells were lysed at 48 hours and luciferase activity was measured. Luciferase units were normalized to 100 in the DMSO condition. (B) HeLa-ACE2 cells were treated with 20 µM of rapamycin (Ra), everolimus (Ev), temsirolimus (Te), ridaforolimus (Ri), or an equivalent volume of DMSO (D) for 4 hours and subsequently exposed to HIV-HA pseudovirus. Cells were lysed at 48 hours and luciferase activity was measured. Luciferase units were normalized to 100 in the DMSO condition. All error bars represent standard error calculated from 3 experiments. Statistical analysis was performed with one-way ANOVA and asterisks indicate significant difference from DMSO. *, $p < 0.05$; **, $p < 0.01$.

bioRxiv preprint doi: https://doi.org/10.1101/2021.04.15.440067; this version posted April 16, 2021. The copyright holder for this preprint (which was not certified by peer review) is the author/funder. This article is a US Government work. It is not subject to copyright under 17 USC 105 and is also made available for use under a CC0 license.

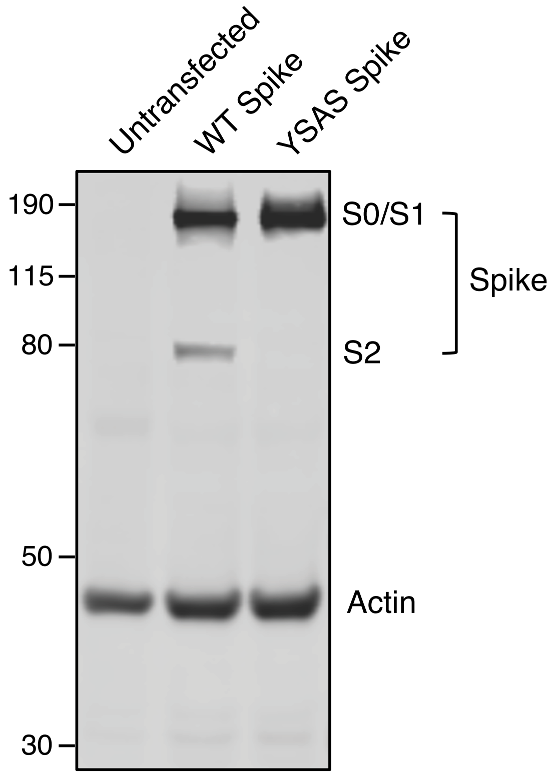

Supplemental Figure 2:
HEK293T cells were transfected with pcDNA3.1 encoding WT SARS-CoV-2 Spike or Spike encoding $_{682}$YSAS$_{685}$ for 48 hours and subsequently subjected to SDS-PAGE and Western blot analysis. Immunoblotting was performed with anti-Spike and anti-actin. Numbers and tick marks indicate size (kilodaltons) and position of protein standards in ladder.

bioRxiv preprint doi: https://doi.org/10.1101/2021.04.15.440067; this version posted April 16, 2021. The copyright holder for this preprint (which was not certified by peer review) is the author/funder. This article is a US Government work. It is not subject to copyright under 17 USC 105 and is also made available for use under a CC0 license.

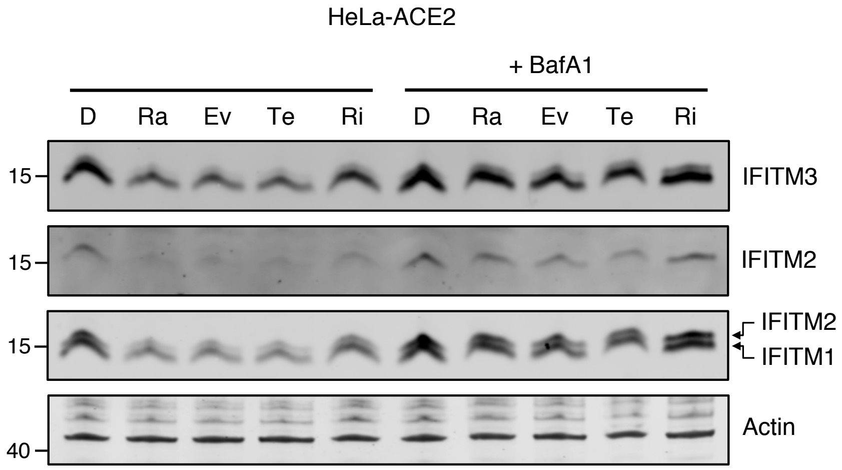

Supplemental Figure 3:
HeLa-ACE2 cells were treated with 20 μM of rapamycin (Ra), everolimus (Ev), temsirolimus (Te), ridaforolimus (Ri), or an equivalent volume of DMSO (D), in the absence or presence of 1 μM bafilomycin A1, for 4 hours. Whole cell lysates were subjected to SDS-PAGE and Western blot analysis with anti-IFITM2, anti-IFITM3, anti-IFITM1, and anti-actin (in that order) on the same nitrocellulose membrane.

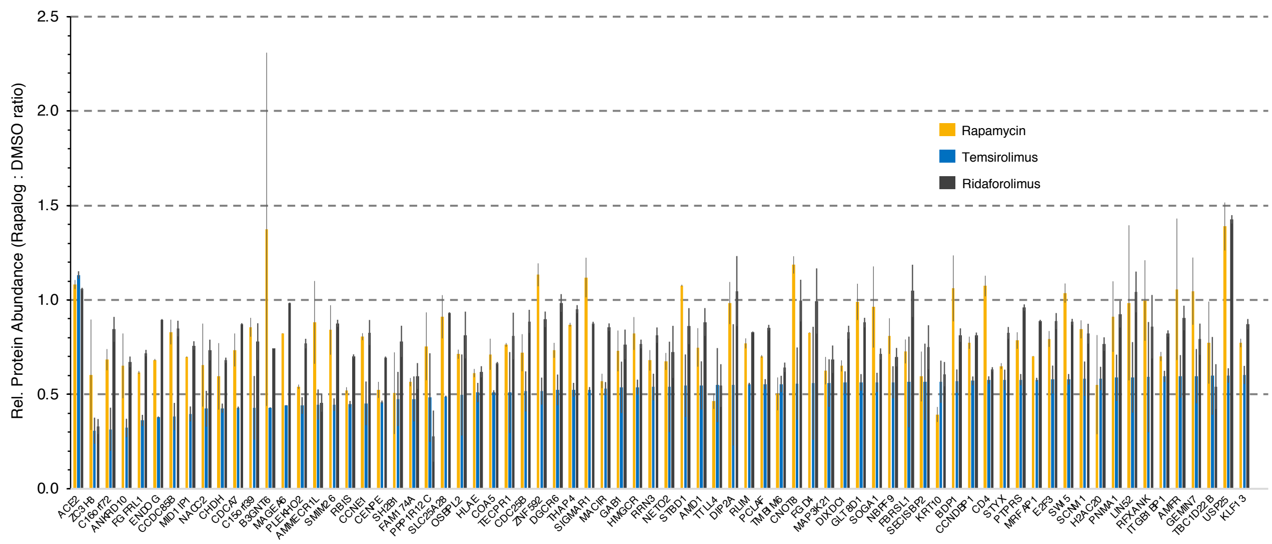

Supplemental Figure 4:
HeLa-ACE2 cells were treated with 20 µM of rapamycin, temsirolimus, ridaforolimus, or an equivalent volume of DMSO for 4 hours and proteins were extracted for analysis by mass spectrometry. 7023 proteins were detected in all conditions and the raw data is presented in Supplemental File 1. The 73 proteins which exhibited a temsirolimus to DMSO ratio of 0.60 or less are shown. ACE2 is also shown for comparison.

Supplemental File 1: Spreadsheet of proteomics data obtained by mass spectrometry.
